# Temporal trends, associated risk factors and longitudinal cardiovascular outcomes of body roundness among middle-aged and older Chinese adults: from the China Health and Retirement Longitudinal Study 2011–2018

**DOI:** 10.3389/fnut.2025.1515067

**Published:** 2025-01-24

**Authors:** Ying-Yuan Gan, Yun-Dan Luo, Lu Zhai, Rong-Rui Huo, Xia Dai, Qian Liao

**Affiliations:** ^1^Department of Scientific Research, Minzu Hospital of Guangxi Zhuang Autonomous Region, Nanning, China; ^2^Department of General Practice, Minzu Hospital of Guangxi Zhuang Autonomous Region, Nanning, China; ^3^Department of Smart Health Elderly Care Services and Management, Guangxi Health Science College, School of Nursing, Nanning, China; ^4^Department of Experimental Research, Guangxi Medical University Cancer Hospital, Nanning, China; ^5^Department of Endocrinology, The First Affiliated Hospital of Guangxi Medical University, Nanning, China; ^6^Department of Epidemiology and Health Statistics, School of Public Health, Guangxi Medical University, Nanning, China

**Keywords:** obesity, cardiovascular disease, China, cohort study, body roundness index

## Abstract

**Background:**

Obesity is a major global health issue, driving high morbidity and mortality rates. The body roundness index (BRI), which includes waist circumference, offers a more accurate measure of visceral and total body fat. However, despite evidence of BRI's effectiveness in predicting obesity-related diseases, national-level data, especially from non-Western populations like China, remain limited.

**Methods:**

This study utilized data from the China Health and Retirement Longitudinal Study (CHARLS), a large, nationally representative cohort of Chinese adults, to examine the temporal trends of BRI, identify associated risk factors, and investigate the longitudinal associations between BRI and cardiovascular disease (CVD) outcomes. BRI was calculated using height and waist circumference measurements. Temporal trends and risk factors were analyzed cross-sectionally, while longitudinal associations were examined using Cox proportional hazards models adjusted for confounders. Mediation analyses were conducted to assess the role of intermediate factors such as hypertension and diabetes in the relationship between BRI and CVD.

**Results:**

A total of 12,902 participants were included for risk factor analysis, 10,525 for longitudinal analysis, and 7,310 for cumulative analysis. BRI continued to rise slowly across survey cycles but was higher in women, older adults, and urban residents. Multivariable analysis identified age, alcohol consumption, elevated blood pressure, and diabetes as positive predictors of BRI, while male sex, rural residence, and smoking were negatively associated. Higher baseline BRI was significantly associated with increased CVD risk (HR: 1.44, 95% CI: 1.22–1.69), stroke (HR: 1.49, 95% CI: 1.12–1.98), and heart disease (HR: 1.47, 95% CI: 1.22–1.77). Cumulative BRI similarly predicted increased risks of CVD, stroke, and heart disease. Mediation analysis showed that hypertension accounted for 20.69% of the association between BRI and CVD risk.

**Conclusions:**

BRI is a robust predictor of CVD risk. Targeting hypertension and other metabolic conditions could mitigate the elevated CVD risk associated with high BRI in Chinese adults. These findings underscore the importance of incorporating BRI into public health strategies to better manage obesity-related health risks in China.

## 1 Introduction

Obesity, a major global health concern, significantly contributes to increased morbidity and mortality rates worldwide (1). Over one billion individuals are currently classified as obese, positioning obesity as one of the top five risk factors for mortality globally, responsible for approximately five million deaths in 2019 (2, 3). Traditionally, obesity has been measured using body mass index (BMI), calculated as weight in kilograms divided by height in meters squared. While BMI is a widely used metric, it has notable limitations, particularly its inability to differentiate between fat and muscle mass or to accurately reflect fat distribution in the body (4).

Recognizing these limitations, researchers have increasingly turned their attention to measures that more accurately capture body fat distribution, especially visceral fat, which is closely linked to various adverse health outcomes. Among these new measures, the body roundness index (BRI) has emerged as a promising tool for assessing obesity-related risks more comprehensively. BRI was introduced by Thomas et al. (5) as an advanced anthropometric measure that not only considers an individual's weight and height but also incorporates waist circumference, a critical indicator of abdominal fat distribution. BRI is calculated using an elliptical model based on human body shape, which allows it to estimate both visceral fat and total body fat percentages more accurately than BMI (5, 6). The incorporation of waist circumference enables BRI to more effectively capture the nuances of body fat distribution, making it a superior predictor of health risks associated with obesity (5, 7). Several studies have demonstrated the advantages of BRI over BMI and other traditional anthropometric measures. For instance, BRI has been found to be a stronger predictor of cardiometabolic diseases such as diabetes (8, 9) and hypertension (10), kidney disease (11, 12), and even certain types of cancer (13). Moreover, longitudinal research has shown that a higher BRI is significantly associated with increased risks of all-cause mortality and cardiovascular disease-specific mortality (7, 14, 15).

Despite the growing evidence supporting the utility of BRI, there remains a paucity of national data examining its trends and associations with long-term health outcomes, particularly in non-Western populations. This gap in the literature is especially relevant in China, where rapid economic development and urbanization have led to significant lifestyle changes and a rising prevalence of obesity (16, 17). Understanding the temporal trends of BRI in the Chinese population, as well as the associated risk factors and longitudinal cardiovascular outcomes, is crucial for developing effective public health strategies to combat the obesity epidemic.

In this study, we leveraged data from a large, nationally representative cohort of Chinese adults to pursue the following objectives: (1) assess the temporal trends in BRI across different demographic groups; (2) identify the risk factors associated with variations in BRI; and (3) investigate the longitudinal associations between BRI and cardiovascular outcomes and reveal the potential mechanisms. Our study aims to fill critical knowledge gaps and contribute to more precise risk stratification and prevention strategies for obesity-related diseases in China and globally.

## 2 Methods

### 2.1 Study procedure and participants

This cohort study conducted a secondary analysis of data from the China Health and Retirement Longitudinal Study (CHARLS), an ongoing national survey. Detailed descriptions of the study design are available in previous publications (18). Between June 2011 and March 2012, 17,708 individuals from 10,257 households in 150 counties and 450 villages across 28 provinces were recruited. A multistage stratified sampling method proportional to population size was utilized. Participants were asked to complete a standardized questionnaire that collected information on sociodemographic characteristics, lifestyle behaviors, and health conditions through face-to-face interviews and health assessments. The response rate for the initial survey was 80.5%. Follow-up surveys were conducted biennially.

**Figure 1:**
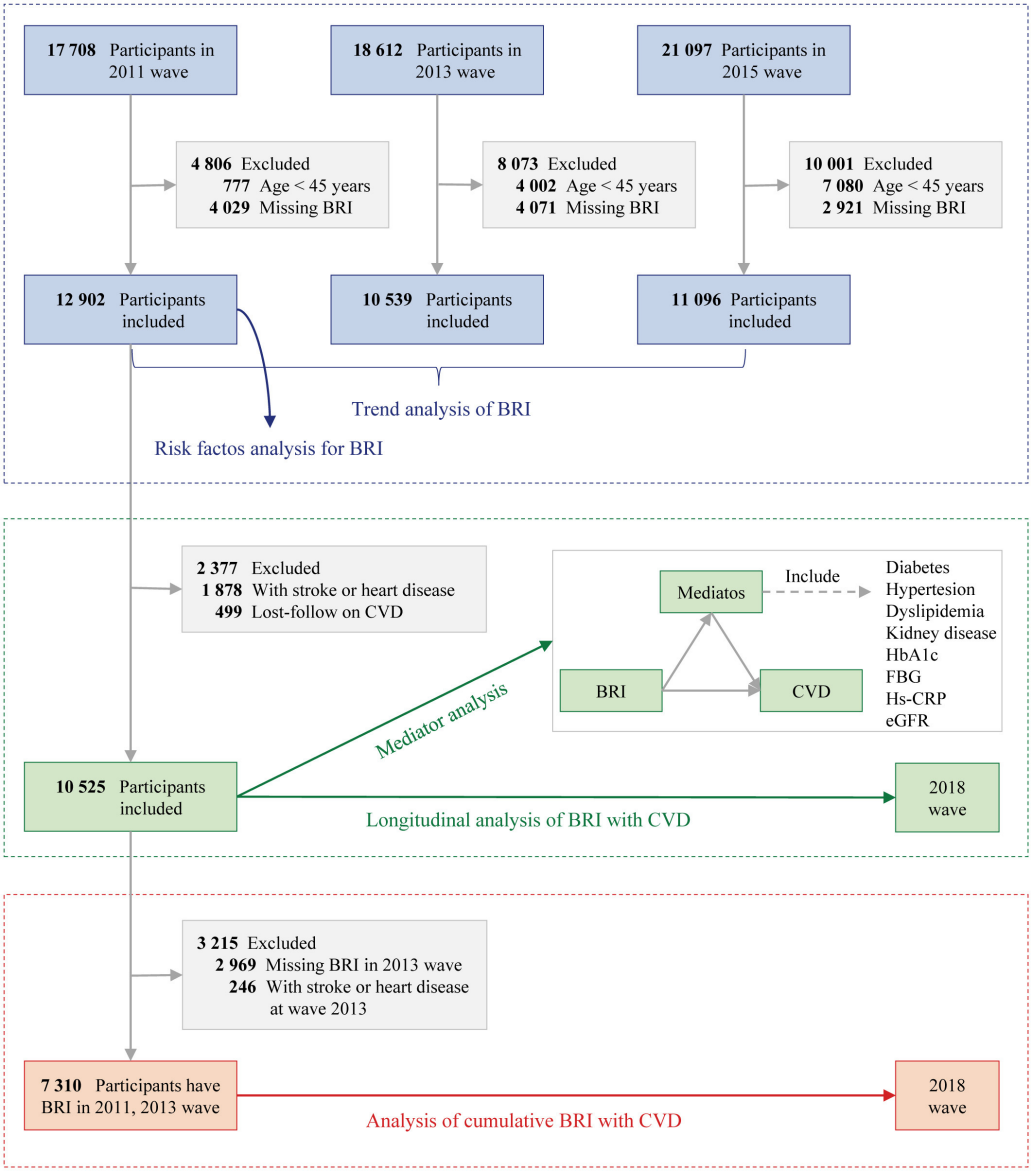
Study flowchart. BRI, Body roundness index; CVD, Cardiovascular disease; eGFR, Estimated glomerular filtration rate; FBG, Fasting plasma glucose; hs-CRP, High-density C-reactive protein; HbA1c, Glycated hemoglobin.

The present study was divided into three parts (Figure 1):

(1) Cross-sectional analysis: Data from the baseline survey (2011 wave), first follow-up (2013), and second follow-up (2015 wave) were used to estimate BRI trends. Participants without BRI data or aged <45 were excluded. Additionally, the baseline survey (2011 wave) was used to assess the associations between baseline risk factors and BRI.
(2) Longitudinal analysis: Participants from the baseline survey (2011 wave) were followed up with data from the final interview (2018 wave). Participants with baseline heart disease, stroke, or missing follow-up CVD data were excluded. Mediating factors (e.g., diabetes, hypertension, dyslipidemia) between BRI and CVD were analyzed.
(3) Cumulative analysis: Cumulative BRI was calculated using data from the baseline (2011 wave) and first follow-up (2013 wave), with follow-up data from the final interview (2018 wave). Participants with heart disease, stroke, or missing BRI data from 2013 were excluded.

Ethical approval for the CHARLS project was granted by Peking University's institutional review board (IRB00001052-11015), and all participants provided written informed consent. The study adhered to the Strengthening the Reporting of Observational Studies in Epidemiology (STROBE) guidelines for observational research (19).

### 2.2 Assessment of BRI

A trained nurse measured body height and WC in the 2011 and 2013 waves. BRI was calculated as 364.2 – 365.5 × √(1 - [WC in centimeters / 2π]^2^/ [0.5 × height in centimeters]^2^), according to the formula developed by Thomas et al. (5). Cumulative BRI was calculated as (BRI_2011_+ BRI_2013_)/2 × time (2013–2011). Due to the lack of a reference range, BRI and cumulative BRI were split into quintiles to explore the associations with CVD.

### 2.3 Assessment of CVD

The primary outcome of the study was CVD. Consistent with prior research (20, 21), CVDs were identified using standardized questions such as: “Has a doctor ever informed you of a diagnosis of a heart attack, coronary heart disease, angina, congestive heart failure, or other heart conditions?” or “Has a doctor ever informed you of a diagnosis of a stroke?” Participants who reported a diagnosis of heart disease or stroke during the follow-up were classified as having experienced CVD.

### 2.4 Covariates

Covariates were collected on sociodemographic status (age, sex, living residence, marital status, and educational level), health-related factors (self-reported smoking and drinking status, self-reported diabetes, hypertension, dyslipidemia, and chronic kidney disease), and laboratory examination (estimated glomerular filtration rate, fasting plasma glucose, high-density C-reactive protein, glycated hemoglobin, high-density lipoprotein cholesterol, low-density lipoprotein cholesterol, total cholesterol, and triglyceride).

Diabetes was defined by fasting plasma glucose levels of 126 mg/dL or higher, current use of antidiabetic medication, or self-reported history of diabetes. Hypertension was identified as systolic blood pressure of 140 mm Hg or greater, diastolic blood pressure of 90 mm Hg or higher, current use of antihypertensive medication, or a self-reported history of hypertension. Dyslipidemia was characterized by total cholesterol levels of 240 mg/dL or above, triglycerides of 150 mg/dL or higher, low-density lipoprotein cholesterol of 160 mg/dL or more, high-density lipoprotein cholesterol below 40 mg/dL, current use of lipid-lowering medication, or self-reported history of dyslipidemia. Chronic kidney disease was defined as an estimated glomerular filtration rate below 60 mL/min/1.73 m^2^ or a self-reported history of the condition. The estimated glomerular filtration rate was calculated using the Chronic Kidney Disease Epidemiology Collaboration's 2009 creatinine equation (22).

### 2.5 Statistical analysis

For descriptive statistics, categorical variables were reported as frequencies with percentages, and continuous variables as means ± SD or medians (interquartile range). Analyses were conducted using the chi-square test, one-way ANOVA, or the Kruskal-Wallis H test, as appropriate. The missing rates of covariates were summarized in Supplementary Tables S1–S3. The missing data of covariates were imputed using the multiple imputation with chained equation.

To account for the study's multistage, stratified, probability-proportional-to-size sampling design, results were weighted to provide nationally representative estimates for non-institutionalized civilian residents of China. Weighted mean BRIs and SDs for 2011, 2013, and 2015 were calculated and compared across subgroups, with trends assessed using partial Mann-Kendall tests. Risk factors for BRI were initially estimated using a generalized linear regression model. Variance inflation factor (VIF) was computed to test for multicollinearity among covariates. TC and LDL-C variables with VIF >10 were excluded from the final model (Supplementary Table S4). Variables with *P* < 0.05 in univariable analysis were also excluded.

To analyze the associations between baseline and cumulative BRI with the risk of incident CVD, both measures were divided into quintiles and included in Cox proportional hazards models to estimate hazard ratios (HR) and 95% confidence intervals (CI), using quintile 1 as the reference. Three models were fitted: Model 1 adjusted for age and sex; Model 2 for age, sex, marital status, residence, education, smoking, and drinking status; and Model 3 for the same variables as Model 2, plus systolic and diastolic blood pressure, diabetes, hypertension, dyslipidemia, kidney disease, glycated hemoglobin, fasting plasma glucose, total cholesterol, triglycerides, high-density lipoprotein cholesterol, low-density lipoprotein cholesterol, high-sensitivity C-reactive protein, and estimated glomerular filtration rate. The assumption of proportional hazards was confirmed via Schoenfeld residuals. Additionally, a restricted cubic spline (RCS) curve with four knots was used to assess nonlinearity in the association between BRI and CVD risk. Subgroup analyses explored whether the associations were moderated by covariates, with interaction terms and likelihood ratio tests used to evaluate interaction effects.

Mediation analysis of the association between baseline BRI (exposure) and CVD (outcome) through mediators such as diabetes, hypertension, dyslipidemia, kidney disease, glycated hemoglobin, fasting plasma glucose, high-sensitivity C-reactive protein, or estimated glomerular filtration rate was conducted using the two-stage regression method for survival data, as proposed by VanderWeele (23). Briefly, two regression models were fitted: one for the mediator and one for the outcome. Parameter estimates and standard errors from these models were combined, following VanderWeele's formulas, to estimate the mediation effect size.

Sensitivity analysis was conducted using the complete data set without multiple imputations. All analyses were conducted using R version 3.5.2 (R Project for Statistical Computing). *P* values were 2-tailed, and *P* < 0.05 was considered statistically significant.

## 3 Results

### 3.1 Characteristics of the study participants

Based on the inclusion and exclusion criteria, 12,902 participants were included for risk factor analysis (47.7% men, mean age: 59.23 ± 9.54 years), 10,525 for longitudinal analysis (48.3% men, mean age: 58.56 ± 9.31 years), and 7,310 for cumulative analysis (48.1% men, mean age: 58.78±9.15 years). The baseline characteristics of these participants are detailed in Table 1. In addition, we described the baseline characteristics of participants using data not being imputed (see Supplementary Tables S5–S7). These results were similar with Table 1.

**Table 1:** Baseline characteritics of study participants.^a^

**Characteritics**	**For risk factors analysis at year 2011 (*n* = 12,902)**	**For longitudinal analysis at year 2011 (*n* = 10,525)**	**For cumulative analysis at year 2011–2013 (*n* = 7,310)**
Age (years)	59.23 ± 9.54	58.56 ± 9.31	58.78 ± 9.15
Age ≥60 years	5,702 (44.2%)	4,335 (41.2%)	3,129 (42.8%)
Men	6,153 (47.7%)	5,083 (48.3%)	3,513 (48.1%)
Married	10,609 (82.2%)	8,751 (83.1%)	6,147 (84.1%)
Rural residence	8,152 (63.2%)	6,889 (65.5%)	4,944 (67.6%)
**Education level**			
No formal education	3,709 (28.7%)	3,047 (29.0%)	2,101 (28.7%)
Primary school	5,220 (40.5%)	4,245 (40.3%)	3,076 (42.1%)
Middle or high school	3,517 (27.3%)	2,898 (27.5%)	1,931 (26.4%)
College or above	456 (3.5%)	335 (3.2%)	202 (2.8%)
**Smoking status**			
Never	7,730 (59.9%)	6,315 (60.0%)	4,402 (60.2%)
Former	1,160 (9.0%)	828 (7.9%)	577 (7.9%)
Current	4,012 (31.1%)	3,382 (32.1%)	2,331 (31.9%)
**Drinking status**			
Never	7,601 (58.9%)	6,120 (58.1%)	4,274 (58.5%)
Former	1,096 (8.5%)	815 (7.7%)	576 (7.9%)
Current	4,205 (32.6%)	3,590 (34.1%)	2,460 (33.7%)
SBP (mmHg)	129.89 ± 21.53	128.90 ± 21.04	128.58 ± 20.77
DBP (mmHg)	75.55 ± 12.25	75.19 ± 12.06	74.88 ± 11.99
Diabetes	1,777 (13.8%)	1,301 (12.4%)	934 (12.8%)
Hypertension	5,373 (41.6%)	3,999 (38.0%)	2,728 (37.3%)
Dyslipidemia	4,898 (38.0%)	3,811 (36.2%)	2,698 (36.9%)
Kidney disease	731 (5.7%)	497 (4.7%)	347 (4.7%)
HbA1c (%)	5.25 ± 0.77	5.22 ± 0.75	5.23 ± 0.74
FBG (mg/dl)	108.59 ± 35.18	107.80 ± 33.15	108.02 ± 33.07
TC (mg/dl)	192.58 ± 39.58	192.26 ± 38.94	192.39 ± 38.82
TG (mg/dl)	102.66 (72.57, 148.46)	100.89 (71.68, 145.14)	100.00 (71.68, 144.26)
HDL-C (mg/dl)	52.49 ± 15.77	52.94 ± 15.64	52.96 ± 15.62
LDL-C (mg/dl)	115.40 ± 35.53	115.06 ± 34.81	115.22 ± 34.68
hs-CRP (mg/l)	1.02 (0.55, 2.14)	0.97 (0.53, 2.08)	0.96 (0.53, 1.99)
eGFR (ml/min/1.73 m^2^)	73.12 (53.83, 96.63)	72.91 (53.67, 96.49)	72.46 (53.80, 96.32)

DBP, Diastolic blood pressure; eGFR, Estimated glomerular filtration rate; FBG, Fasting plasma glucose; hs-CRP, High-density C-reactive protein; HbA1c, Glycated hemoglobin; HDL-C, High-density lipoprotein cholesterol; LDL-C, Low-density lipoprotein cholesterol; SBP, Systolic blood pressure; TC, Total cholesterol; TG, Triglyceride.

^a^data were mean ± SD, median (IQR) or n(%), unless otherwise specified.

### 3.2 Temporal trends of BRI

**Figure 2:**
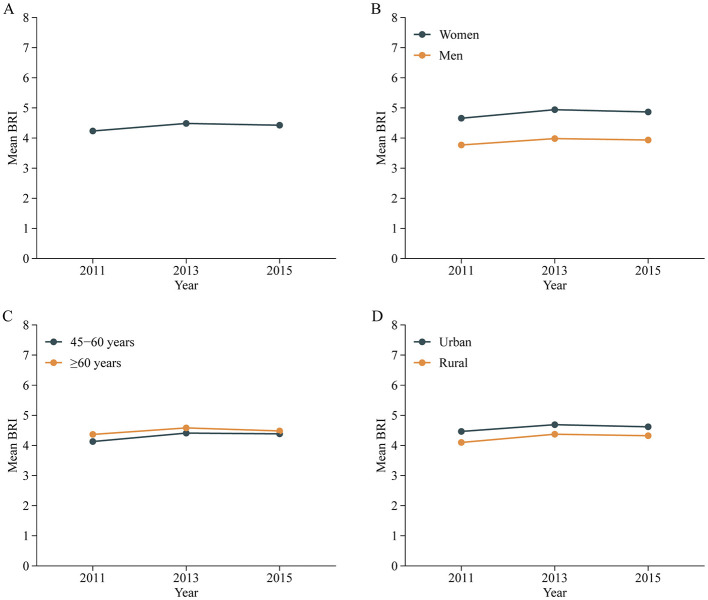
Trends of mean BRI values in all participants **(A)** and stratified by sex **(B)**, age **(C)** and residence **(D)** in Chinese adults. Nationally representative estimates of the Chinese population aged 45 years or older. Estimates were nationally representative through the use of China Health and Retirement Longitudinal Study (CHARLS). BRI, Body roundness index.

The temporal trends of BRI are presented in Figure 2A and the distribution is presented Supplementary Figure S1. The mean BRI values for 2011, 2013, and 2015 were 4.23 ± 1.40, 4.48 ± 1.47, and 4.43 ± 1.47, respectively, indicating a stable trend over time (P for trend < 0.001). BRI trends across CHARLS cycles, stratified by sociodemographic factors, remained generally consistent. Specifically, BRI was higher in women than men (Figure 2B). Age-group analysis (Figure 2C) showed that BRI increased with age and across cycles, although the difference between elderly participants (≥60 years) and middle-aged counterparts (45–60 years) was minimal. Additionally, BRI was higher in urban compared to rural areas (Figure 2D).

### 3.3 Associated factors for BRI

A total of 12,902 adults were included in this analysis. Multivariable analysis (Table 2) indicates that older age (≥60 years), current alcohol consumption, elevated SBP, DBP, HbA1c, TG, and the presence of chronic conditions (e.g., diabetes, hypertension, stroke, heart disease) are positively associated with BRI. In contrast, male sex, rural residence, current smoking, and higher levels of FBG and HDL-C are negatively associated with BRI.

**Table 2:** Factors associated with body roundness index in 2011.

**Factors**	**Univariable analysis**		**Multivariable analysis**	
	β **(95% CI)**	***P*** **value**	β **(95% CI)**	***P*** **value**
**Age (years)**				
45-60	0 (Reference)		0 (Reference)	
≥60	0.22 (0.17 to 0.27)	< 0.001	0.16 (0.11 to 0.22)	< 0.001
**Sex**				
Women	0 (Reference)		0 (Reference)	
Men	−0.88 (−0.93 to −0.83)	< 0.001	−0.77 (−0.85 to −0.70)	< 0.001
**Marital status**				
Other	0 (Reference)			
Married	−0.07 (−0.14 to 0.00)	0.056		
**Residence**				
Urban	0 (Reference)		0 (Reference)	
Rural	−0.33 (−0.39 to −0.28)	< 0.001	−0.22 (−0.27 to −0.17)	< 0.001
**Education level**				
No formal education	0 (Reference)		0 (Reference)	
Primary school	−0.32 (−0.38 to −0.26)	< 0.001	−0.01 (−0.07 to 0.05)	0.777
Middle or high school	−0.37 (−0.44 to −0.30)	< 0.001	0.01 (−0.06 to 0.08)	0.844
College or above	−0.33 (−0.48 to −0.17)	< 0.001	−0.08 (−0.21 to 0.06)	0.293
**Smoking status**				
Never	0 (Reference)		0 (Reference)	
Former	−0.37 (−0.47 to −0.28)	< 0.001	0.03 (−0.07 to 0.13)	0.550
Current	−0.81 (−0.86 to −0.75)	< 0.001	−0.27 (−0.33 to −0.20)	< 0.001
**Drinking status**				
Never	0 (Reference)		0 (Reference)	
Former	−0.24 (−0.34 to −0.14)	< 0.001	0.06 (−0.03 to 0.15)	0.174
Current	−0.51 (−0.57 to −0.45)	< 0.001	0.07 (0.01 to 0.13)	0.024
SBP (mmHg), per 1-SD increment	0.32 (0.29 to 0.34)	< 0.001	0.11 (0.07 to 0.15)	< 0.001
DBP (mmHg), per 1-SD increment	0.25 (0.23 to 0.28)	< 0.001	0.08 (0.05 to 0.12)	< 0.001
**Diabetes**				
No	0 (Reference)		0 (Reference)	
Yes	0.54 (0.46 to 0.61)	< 0.001	0.20 (0.11 to 0.29)	< 0.001
**Hypertension**				
No	0 (Reference)		0 (Reference)	
Yes	0.70 (0.65 to 0.75)	< 0.001	0.28 (0.21 to 0.34)	< 0.001
**Dyslipidemia**				
No	0 (Reference)		0 (Reference)	
Yes	0.61 (0.56 to 0.66)	< 0.001	0.26 (0.21 to 0.32)	< 0.001
**Kidney disease**				
No	0 (Reference)			
Yes	−0.11 (−0.23 to 0.01)	0.075		
**Stroke**				
No	0 (Reference)		0 (Reference)	
Yes	0.44 (0.33 to 0.55)	< 0.001	0.19 (0.09 to 0.28)	< 0.001
**Heart disease**				
No	0 (Reference)		0 (Reference)	
Yes	0.40 (0.33 to 0.48)	< 0.001	0.15 (0.08 to 0.21)	< 0.001
HbA1c (%), per 1-SD increment	0.17 (0.14 to 0.19)	< 0.001	0.11 (0.08 to 0.14)	< 0.001
FBG (mg/dl), per 1-SD increment	0.14 (0.12 to 0.17)	< 0.001	−0.07 (−0.10 to −0.03)	< 0.001
TC (mg/dl), per 1-SD increment	0.16 (0.14 to 0.19)	< 0.001		
TG (mg/dl), per 1-SD increment	0.24 (0.21 to 0.27)	< 0.001	0.06 (0.04 to 0.09)	< 0.001
HDL-C (mg/dl), per 1-SD increment	−0.26 (−0.28 to −0.23)	< 0.001	−0.15 (−0.18 to −0.13)	< 0.001
LDL-C (mg/dl), per 1-SD increment	0.14 (0.11 to 0.16)	< 0.001		
hs-CRP (mg/l), per 1-SD increment	0.03 (0.01 to 0.06)	0.014	0.01 (−0.01 to 0.04)	0.220
eGFR (ml/min/1.73 m^2^), per 1-SD increment	−0.14 (−0.17 to −0.12)	< 0.001	0.02 (−0.01 to 0.04)	0.203

CI, Confidence interval; DBP, Diastolic blood pressure; eGFR, Estimated glomerular filtration rate; FBG, Fasting plasma glucose; hs-CRP, High-density C-reactive protein; HbA1c, Glycated hemoglobin; HDL-C, High-density lipoprotein cholesterol; LDL-C, Low-density lipoprotein cholesterol; SBP, Systolic blood pressure; TC, Total cholesterol; TG, Triglyceride.

### 3.4 Longitudinal associations between baseline BRI with CVD

**Figure 3:**
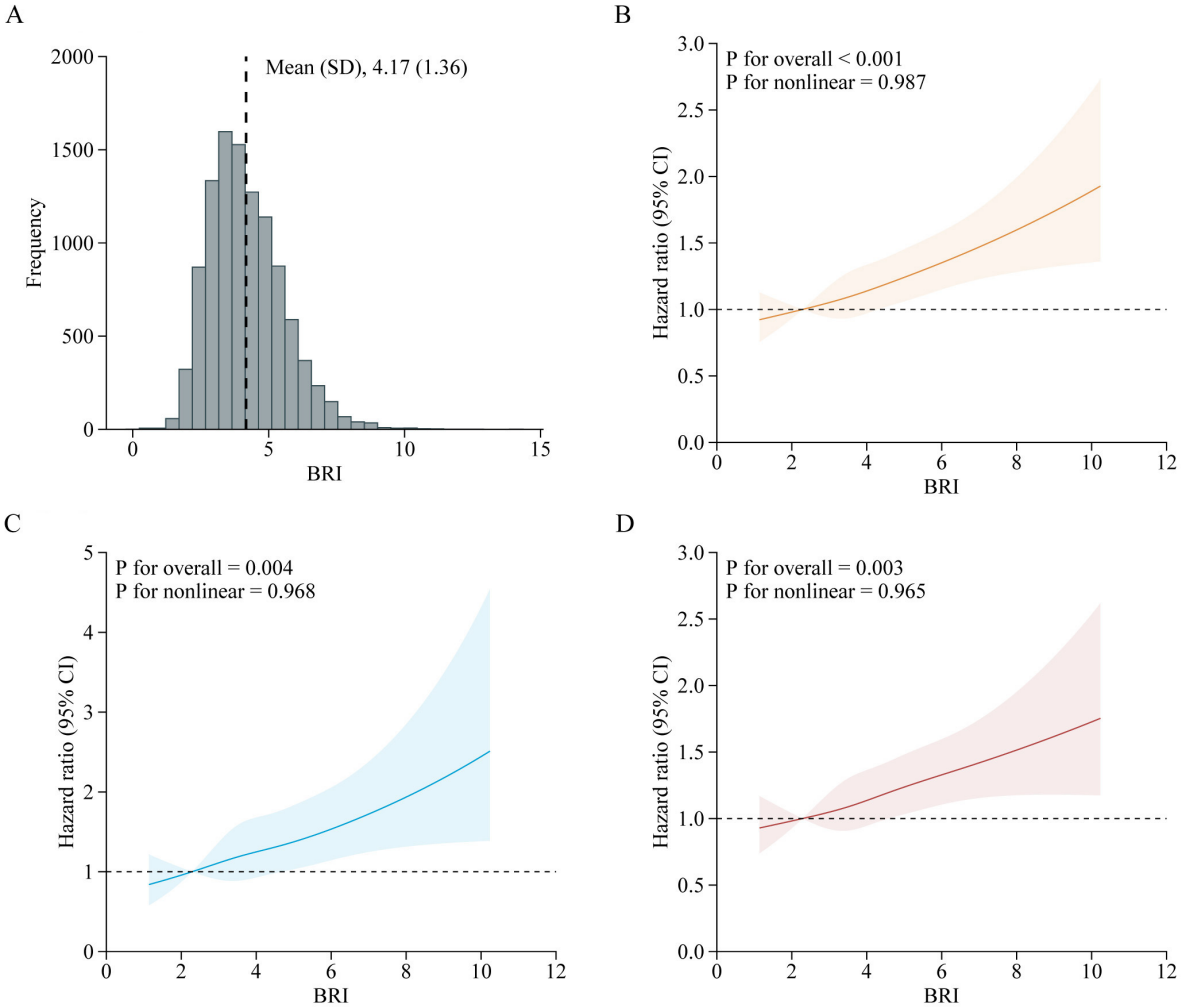
Adjusted HRs of CVD events risk according to baseline BRI. **(A)** Distribution for BRI in all participants; **(B–D)** Graphs show HRs for CVD **(B)**, stroke **(C)**, and heart disease **(D)** adjusted for age, sex, marital status, residence, education level, smoking status, drinking status, systole blood pressure, diastolic blood pressure, diabetes, hypertension, dyslipidemia, kidney disease, glycated hemoglobin, fasting plasma glucose, total cholesterol, triglycerides, high-density lipoprotein cholesterol, low-density lipoprotein cholesterol, high-sensitivity c-reactive protein, and estimated glomerular filtration rate. Data were fitted by a restricted cubic spline Cox proportional hazards regression model. Solid lines indicate HRs, and shadow shapes indicate 95% CIs. BRI, Body roundness index; CVD, Cardiovascular disease; CI, Confidence interval; HR, Hazard ratio.

**Figure 4:**
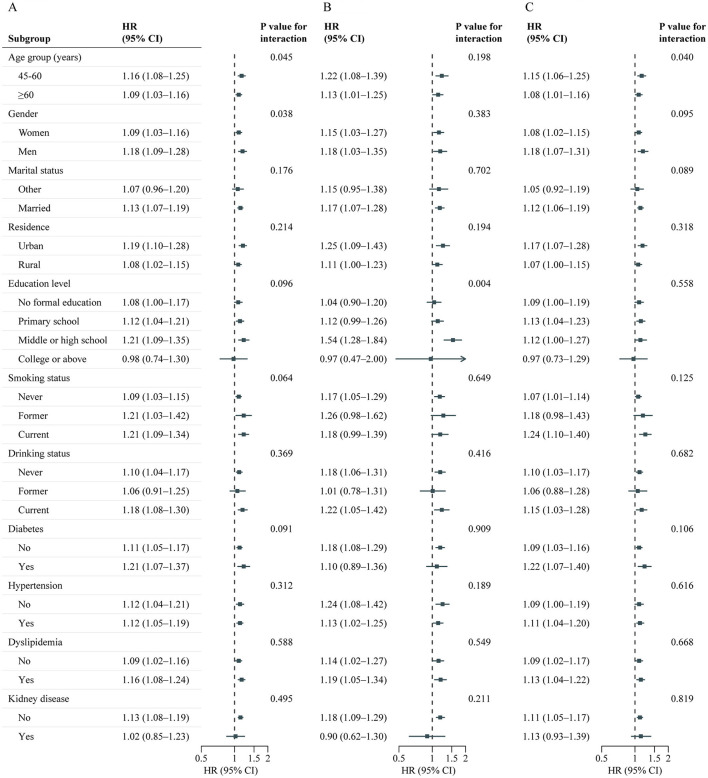
Association between baseline BRI and CVD events risk stratified by different factors. Graphs show HRs and 95% CIs for CVD **(A)**, heart disease **(B)**, and stroke **(C)** according to per 1-SD increment of baseline BRI adjusted for age, sex, marital status, residence, education level, smoking status, drinking status, systole blood pressure, diastolic blood pressure, diabetes, hypertension, dyslipidemia, kidney disease, glycated hemoglobin, fasting plasma glucose, total cholesterol, triglycerides, high-density lipoprotein cholesterol, low-density lipoprotein cholesterol, high-sensitivity c-reactive protein, and estimated glomerular filtration rate. BRI, Body roundness index; CVD, Cardiovascular disease; CI, Confidence interval; HR, Hazard ratio.

A total of 10,525 adults were included in this analysis. Supplementary Table S8 presents participant characteristics by baseline BRI quintiles. During the follow-up period from 2011 to 2018, 1,994 participants developed incident CVD (642 cases of stroke; 1,518 cases of heart disease), with an incidence rate of 18.9% (6.1% cases of stroke; 14.4% cases of heart disease). Table 3 details the associations between baseline BRI and incident CVD. Comparing quintile 5 to quintile 1, the adjusted HRs were 1.44 (95% CI, 1.22–1.69) for CVD, 1.49 (95% CI, 1.12–1.98) for stroke, and 1.47 (95% CI, 1.22–1.77) for heart disease after adjusting for confounders. RCS regression revealed a linear, positive association between baseline BRI and incident CVD risk (P for nonlinearity: CVD, 0.987; stroke, 0.968; heart disease, 0.965) (Figure 3). Each SD increase in baseline BRI was associated with a 12.0% increase in CVD risk (HR: 1.12, 95% CI: 1.07–1.17), a 16.0% increase in stroke risk (HR: 1.16, 95% CI: 1.07–1.26), and an 11.0% increase in heart disease risk (HR: 1.11, 95% CI: 1.05–1.17) (Table 3). Figure 4 presents the association between baseline BRI and incident CVD events, stratified by potential risk factors. The association was more pronounced among middle-aged participants compared to elderly participants (P for interaction = 0.045) and among men compared to women (*P* for interaction = 0.038).

**Table 3:** Longitudinal associations between baseline BRI and CVD.

**Outcomes**	**No. of event/total**	**Model 1** ^**a**^		**Model 2** ^**b**^		**Model 3** ^**c**^	
		**HR (95% CI)**	***P*** **value**	**HR (95% CI)**	***P*** **value**	**HR (95% CI)**	***P*** **value**
**CVD**							
**BRI, quintiles**							
Quintile 1	280/2,105	1 (Reference)		1 (Reference)		1 (Reference)	
Quintile 2	351/2,105	1.26 (1.08–1.48)	0.004	1.26 (1.07–1.47)	0.004	1.19 (1.02–1.39)	0.032
Quintile 3	367/2,105	1.30 (1.11–1.52)	0.001	1.27 (1.09–1.49)	0.003	1.14 (0.98–1.34)	0.098
Quintile 4	433/2,106	1.50 (1.29–1.75)	< 0.001	1.47 (1.26–1.71)	< 0.001	1.23 (1.05–1.44)	0.012
Quintile 5	563/2,104	1.92 (1.65–2.23)	< 0.001	1.88 (1.62–2.19)	< 0.001	1.44 (1.22–1.69)	< 0.001
BRI, per 1-SD increment	1,994/10,525	1.23 (1.18–1.29)	< 0.001	1.22 (1.17–1.28)	< 0.001	1.12 (1.07–1.17)	< 0.001
**Stroke**							
**BRI, quintiles**							
Quintile 1	87/2,105	1 (Reference)		1 (Reference)		1 (Reference)	
Quintile 2	95/2,105	1.12 (0.83–1.49)	0.459	1.12 (0.84–1.50)	0.453	1.04 (0.77–1.39)	0.805
Quintile 3	133/2,105	1.63 (1.24–2.14)	< 0.001	1.62 (1.23–2.13)	0.001	1.37 (1.04–1.81)	0.025
Quintile 4	142/2,106	1.73 (1.32–2.27)	< 0.001	1.72 (1.31–2.26)	< 0.001	1.28 (0.96–1.69)	0.088
Quintile 5	185/2,104	2.31 (1.77–3.02)	< 0.001	2.30 (1.75–3.01)	< 0.001	1.49 (1.12–1.98)	0.006
BRI, per 1-SD increment	642/10,525	1.34 (1.25–1.44)	< 0.001	1.34 (1.24–1.44)	< 0.001	1.16 (1.07–1.26)	< 0.001
**Heart disease**							
**BRI, quintiles**							
Quintile 1	207/2,105	1 (Reference)		1 (Reference)		1 (Reference)	
Quintile 2	277/2,105	1.33 (1.11–1.59)	0.002	1.32 (1.10–1.58)	0.003	1.26 (1.05–1.51)	0.013
Quintile 3	273/2,105	1.26 (1.05–1.51)	0.013	1.22 (1.02–1.47)	0.030	1.13 (0.94–1.35)	0.210
Quintile 4	321/2,106	1.43 (1.20–1.71)	< 0.001	1.39 (1.16–1.66)	< 0.001	1.21 (1.00–1.45)	0.046
Quintile 5	440/2,104	1.87 (1.58–2.23)	< 0.001	1.82 (1.53–2.17)	< 0.001	1.47 (1.22–1.77)	< 0.001
BRI, per 1-SD increment	1,518/10,525	1.20 (1.14–1.26)	< 0.001	1.19 (1.13–1.25)	< 0.001	1.11 (1.05–1.17)	< 0.001

BRI, Body roundness index; CVD, Cardiovascular disease; HR, Hazard ratio.

^a^adjusted for age and sex.

^b^adjusted for age, sex, marital status, residence, education level, smoking status, and drinking status.

^c^adjusted for age, sex, marital status, residence, education level, smoking status, drinking status, systole blood pressure, diastolic blood pressure, diabetes, hypertension, dyslipidemia, kidney disease, glycated hemoglobin, fasting plasma glucose, total cholesterol, triglycerides, high-density lipoprotein cholesterol, low-density lipoprotein cholesterol, high-sensitivity c-reactive protein, and estimated glomerular filtration rate.

### 3.5 Associations between cumulative BRI with CVD

**Figure 5:**
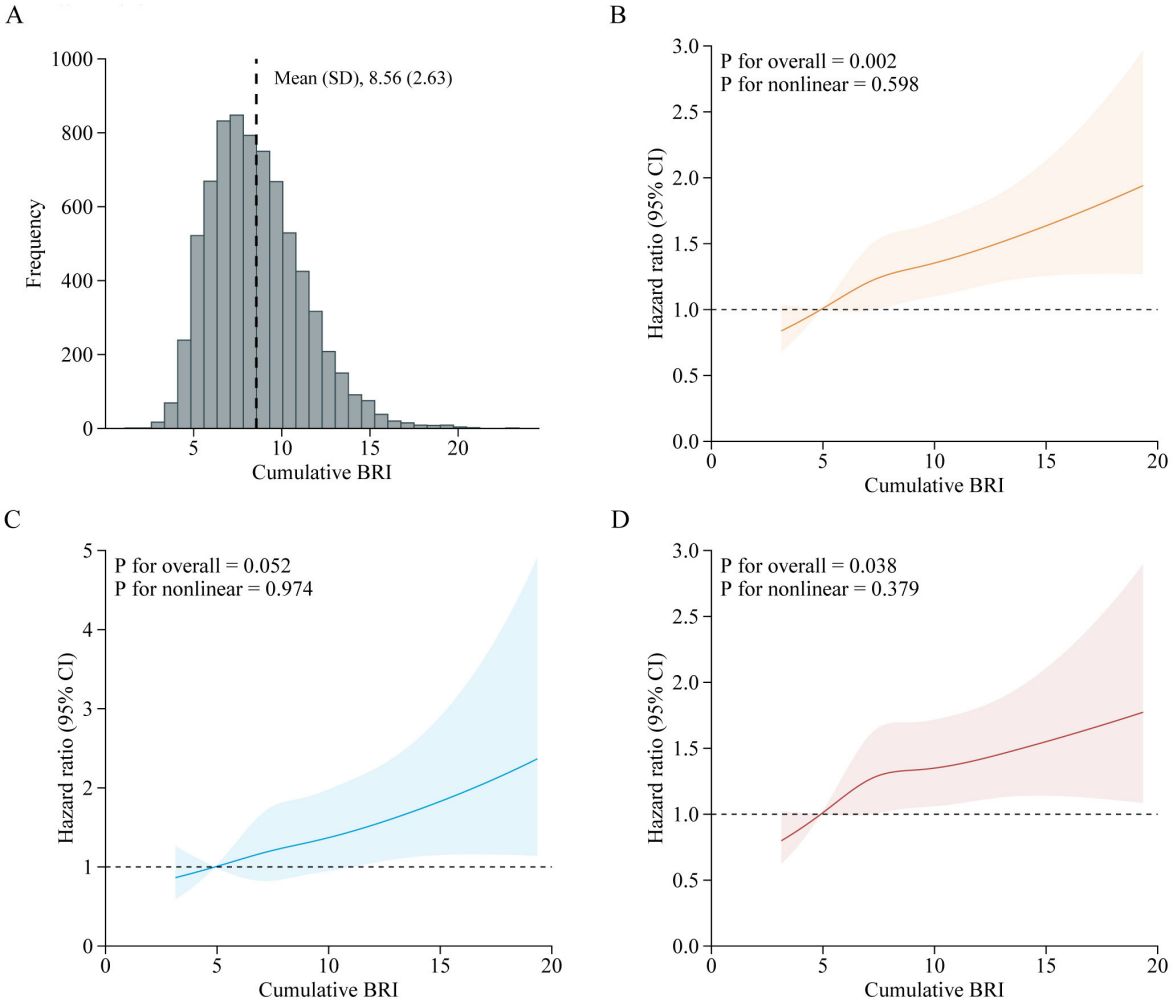
Adjusted HRs of CVD events risk according to cumulative BRI. **(A)** Distribution for cumulative BRI in all participants; **(B–D)** Graphs show HRs for CVD **(B)**, stroke **(C)**, and heart disease **(D)** adjusted for age, sex, marital status, residence, education level, smoking status, drinking status, systole blood pressure, diastolic blood pressure, diabetes, hypertension, dyslipidemia, kidney disease, glycated hemoglobin, fasting plasma glucose, total cholesterol, triglycerides, high-density lipoprotein cholesterol, low-density lipoprotein cholesterol, high-sensitivity c-reactive protein, and estimated glomerular filtration rate. Data were fitted by a restricted cubic spline Cox proportional hazards regression model. Solid lines indicate HRs, and shadow shapes indicate 95% CIs. BRI, Body roundness index; CVD, Cardiovascular disease; CI, Confidence interval; HR, Hazard ratio.

**Figure 6:**
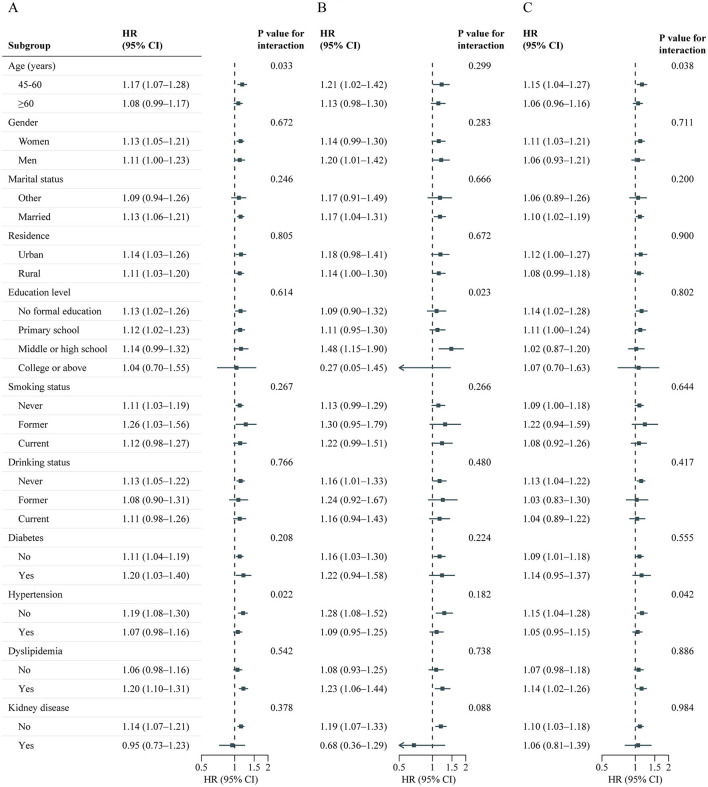
Association between cumulative BRI and CVD events risk stratified by different factors. Graphs show HRs and 95% CIs for CVD **(A)**, heart disease **(B)**, and stroke **(C)** according to per 1-SD increment of cumulative BRI adjusted for age, sex, marital status, residence, education level, smoking status, drinking status, systole blood pressure, diastolic blood pressure, diabetes, hypertension, dyslipidemia, kidney disease, glycated hemoglobin, fasting plasma glucose, total cholesterol, triglycerides, high-density lipoprotein cholesterol, low-density lipoprotein cholesterol, high-sensitivity c-reactive protein, and estimated glomerular filtration rate. BRI, Body roundness index; CVD, Cardiovascular disease; CI, Confidence interval; HR, Hazard ratio.

A total of 7,310 adults were included in this analysis. Supplementary Table S9 presents participant characteristics by cumulative BRI quintiles. Table 4 shows the association between cumulative BRI and incident CVD risk. After adjusting for confounders, participants in the highest quintile of cumulative BRI (quintile 5) had significantly higher risks of incident CVD compared to those in the lowest quintile (quintile 1) (CVD: HR 1.47, 95% CI: 1.20–1.82; stroke: HR 1.53, 95% CI: 1.06–2.22; heart disease: HR 1.44, 95% CI: 1.13–1.83). RCS regression revealed a linear, positive association between cumulative BRI and incident CVD risk (P for nonlinearity: CVD, 0.598; stroke, 0.974; heart disease, 0.379; Figure 5). Each SD increase in cumulative BRI was associated with a 12.0% increase in CVD risk (HR: 1.12, 95% CI: 1.06–1.19), a 16.0% increase in stroke risk (HR: 1.16, 95% CI: 1.05–1.29), and a 10.0% increase in heart disease risk (HR: 1.10, 95% CI: 1.02–1.17; Table 4). The association was more pronounced among middle–aged participants compared to elderly participants (P for interaction = 0.033; Figure 6).

**Table 4:** Associations between cumulative BRI and CVD.

**Outcomes**	**No. of event/total**	**Model 1** ^**a**^		**Model 2** ^**b**^		**Model 3** ^**c**^	
		**HR (95% CI)**	***P*** **value**	**HR (95% CI)**	***P*** **value**	**HR (95% CI)**	***P*** **value**
**CVD**							
**Cumulative BRI, quintiles**							
Quintile 1	169/1,462	1 (Reference)		1 (Reference)		1 (Reference)	
Quintile 2	227/1,462	1.35 (1.10–1.64)	0.004	1.34 (1.10–1.64)	0.004	1.26 (1.03–1.54)	0.024
Quintile 3	238/1,462	1.38 (1.13–1.68)	0.002	1.35 (1.11–1.65)	0.003	1.21 (0.99–1.48)	0.068
Quintile 4	281/1,462	1.65 (1.35–2.00)	< 0.001	1.61 (1.32–1.96)	< 0.001	1.37 (1.12–1.68)	0.003
Quintile 5	347/1,462	1.96 (1.61–2.38)	< 0.001	1.91 (1.57–2.33)	< 0.001	1.47 (1.20–1.82)	< 0.001
Cumulative BRI, per 1-SD increment	1,262/7,310	1.23 (1.16–1.30)	< 0.001	1.22 (1.15–1.29)	< 0.001	1.12 (1.06–1.19)	< 0.001
**Stroke**							
**Cumulative BRI, quintiles**							
Quintile 1	54/1,462	1 (Reference)		1 (Reference)		1 (Reference)	
Quintile 2	65/1,462	1.26 (0.88–1.81)	0.210	1.27 (0.89–1.83)	0.191	1.16 (0.80–1.66)	0.437
Quintile 3	78/1,462	1.53 (1.08–2.17)	0.017	1.54 (1.08–2.18)	0.017	1.27 (0.89–1.82)	0.185
Quintile 4	93/1,462	1.90 (1.35–2.68)	< 0.001	1.92 (1.36–2.72)	< 0.001	1.45 (1.01–2.07)	0.043
Quintile 5	112/1,462	2.33 (1.65–3.28)	< 0.001	2.34 (1.66–3.32)	< 0.001	1.53 (1.06–2.22)	0.024
Cumulative BRI, per 1-SD increment	402/7,310	1.33 (1.21–1.46)	< 0.001	1.32 (1.20–1.46)	< 0.001	1.16 (1.05–1.29)	0.006
**Heart disease**							
**Cumulative BRI, quintiles**							
Quintile 1	124/1,462	1 (Reference)		1 (Reference)		1 (Reference)	
Quintile 2	178/1,462	1.39 (1.11–1.75)	0.005	1.38 (1.10–1.74)	0.006	1.31 (1.04–1.65)	0.022
Quintile 3	181/1,462	1.36 (1.08–1.72)	0.009	1.33 (1.06–1.68)	0.015	1.22 (0.96–1.54)	0.102
Quintile 4	208/1,462	1.54 (1.23–1.94)	< 0.001	1.50 (1.19–1.89)	0.001	1.32 (1.04–1.67)	0.022
Quintile 5	264/1,462	1.83 (1.46–2.29)	< 0.001	1.78 (1.42–2.23)	< 0.001	1.44 (1.13–1.83)	0.003
Cumulative BRI, per 1-SD increment	955/7,310	1.19 (1.11–1.26)	< 0.001	1.18 (1.10–1.25)	< 0.001	1.10 (1.02–1.17)	0.009

BRI, Body roundness; CVD, Cardiovascular disease; HR, Hazard ratio.

^a^adjusted for age and sex.

^b^adjusted for age, sex, marital status, residence, education level, smoking status, and drinking status.

^c^adjusted for age, sex, marital status, residence, education level, smoking status, drinking status, systole blood pressure, diastolic blood pressure, diabetes, hypertension, dyslipidemia, kidney disease, glycated hemoglobin, fasting plasma glucose, total cholesterol, triglycerides, high-density lipoprotein cholesterol, low-density lipoprotein cholesterol, high-sensitivity c-reactive protein, and estimated glomerular filtration rate.

### 3.6 Mediation analysis

As shown in Table 5, significant mediated effects by hypertension, dyslipidemia, HbA1c, FBG, and diabetes were observed on the relationship between BRI and incident CVD risk. Among these, hypertension was the most significant mediator, accounting for 20.69% (95% CI: 13.94–27.44%) of the BRI–associated increased CVD risk (stroke: 21.67%, 95% CI: 13.13–30.21%; heart disease: 22.33%, 95% CI: 12.90–31.77%), followed by dyslipidemia, which mediated 9.49% (95% CI: 5.44–13.53%) of the association (stroke: 9.38%, 95% CI: 4.25–14.50%; heart disease: 9.46%, 95% CI: 4.19–14.73%).

**Table 5:** Mediated proportion on the associations between body roundness index and CVD by different factors.

**Mediators**	**Associations, HR (95% CI)** ^**a**^			**Proportion mediated, % (95% CI)**
	**Total**	**Direct**	**Indirect**	
**CVD**			
Diabetes	1.16 (1.12 to 1.19)	1.15 (1.12 to 1.19)	1.00 (1.00 to 1.00)	1.53 (0.20 to 2.85)
Hypertension	1.14 (1.10 to 1.18)	1.11 (1.07 to 1.15)	1.03 (1.02 to 1.03)	20.69 (13.94 to 27.44)
Dyslipidemia	1.15 (1.11 to 1.19)	1.14 (1.10 to 1.17)	1.01 (1.01 to 1.02)	9.49 (5.44 to 13.53)
Kidney disease	1.16 (1.12 to 1.20)	1.16 (1.12 to 1.20)	1.00 (1.00 to 1.00)	−0.56 (−1.79 to 0.67)
HbA1c (%)	1.16 (1.12 to 1.20)	1.15 (1.12 to 1.19)	1.00 (1.00 to 1.01)	3.18 (0.59 to 5.76)
FBG (mg/dl)	1.16 (1.12 to 1.20)	1.16 (1.12 to 1.19)	1.00 (1.00 to 1.01)	2.37 (0.03 to 4.71)
hs-CRP (mg/l)	1.16 (1.12 to 1.20)	1.16 (1.12 to 1.20)	1.00 (1.00 to 1.00)	0.01 (−0.70 to 0.72)
eGFR (ml/min/1.73 m^2^)	1.16 (1.12 to 1.20)	1.16 (1.12 to 1.20)	1.00 (1.00 to 1.00)	0.12 (−0.21 to 0.46)
**Stroke**			
Diabetes	1.23 (1.17 to 1.30)	1.23 (1.16 to 1.30)	1.00 (1.00 to 1.01)	1.74 (0.02 to 3.46)
Hypertension	1.21 (1.15 to 1.28)	1.17 (1.10 to 1.23)	1.04 (1.03 to 1.05)	21.67 (13.13 to 30.21)
Dyslipidemia	1.23 (1.16 to 1.30)	1.20 (1.14 to 1.27)	1.02 (1.01 to 1.03)	9.38 (4.25 to 14.50)
Kidney disease	1.24 (1.17 to 1.31)	1.24 (1.17 to 1.31)	1.00 (1.00 to 1.00)	−0.08 (−0.50 to 0.34)
HbA1c (%)	1.24 (1.17 to 1.31)	1.23 (1.16 to 1.30)	1.01 (1.00 to 1.01)	4.21 (1.20 to 7.23)
FBG (mg/dl)	1.24 (1.17 to 1.31)	1.23 (1.16 to 1.30)	1.01 (1.00 to 1.01)	3.91 (1.28 to 6.53)
hs-CRP (mg/l)	1.24 (1.17 to 1.31)	1.24 (1.17 to 1.31)	1.00 (1.00 to 1.00)	0.16 (−0.66 to 0.98)
eGFR (ml/min/1.73 m^2^)	1.24 (1.17 to 1.31)	1.24 (1.17 to 1.31)	1.00 (1.00 to 1.00)	0.18 (−0.15 to 0.50)
**Heart disease**			
Diabetes	1.13 (1.09 to 1.18)	1.13 (1.09 to 1.17)	1.00 (1.00 to 1.00)	1.55 (−0.18 to 3.28)
Hypertension	1.12 (1.08 to 1.16)	1.09 (1.05 to 1.13)	1.02 (1.02 to 1.03)	22.33 (12.90 to 31.77)
Dyslipidemia	1.13 (1.09 to 1.17)	1.12 (1.08 to 1.16)	1.01 (1.01 to 1.02)	9.46 (4.19 to 14.73)
Kidney disease	1.14 (1.10 to 1.18)	1.14 (1.10 to 1.18)	1.00 (1.00 to 1.00)	−0.82 (−2.61 to 0.98)
HbA1c (%)	1.14 (1.10 to 1.18)	1.13 (1.09 to 1.18)	1.00 (1.00 to 1.01)	2.20 (−1.20 to 5.60)
FBG (mg/dl)	1.14 (1.10 to 1.18)	1.13 (1.09 to 1.18)	1.00 (1.00 to 1.01)	1.49 (−1.66 to 4.64)
hs-CRP (mg/l)	1.14 (1.10 to 1.18)	1.14 (1.10 to 1.18)	1.00 (1.00 to 1.00)	−0.01 (−0.96 to 0.95)
eGFR (ml/min/1.73 m^2^)	1.14 (1.10 to 1.18)	1.14 (1.10 to 1.18)	1.00 (1.00 to 1.00)	0.18 (−0.27 to 0.64)

CVD, Cardiovascular disease; eGFR, Estimated glomerular filtration rate; FBG, Fasting plasma glucose; hs-CRP, High-density C-reactive protein; HbA1c, Glycated hemoglobin; HR, Hazard ratio. ^a^adjusted for age, sex, marital status, residence, education level, smoking status, and drinking status.

### 3.7 Sensitivity analysis

Similar results were obtained from the complete data analysis on associated factors for BRI (Supplementary Table S10), associations between baseline BRI and CVD (Supplementary Table S11), associations between cumulative BRI and CVD (Supplementary Table S12), and mediation analysis (Supplementary Table S13).

## 4 Discussion

This cohort study examined temporal trends in the BRI among Chinese adults aged 45 years and older from 2011 to 2015, identifying key risk factors and associations with cardiovascular outcomes. BRI increased over the study period, with more pronounced rises in women, older adults, and urban residents. Multivariable analysis revealed that factors such as older age, alcohol consumption, and elevated blood pressure and HbA1c were positively associated with higher BRI. Both baseline and cumulative BRI values were linked to increased risks of CVD, stroke, and heart disease, with mediation analysis highlighting the role of hypertension and dyslipidemia in the BRI-CVD relationship.

Obesity, particularly visceral obesity, is a well-established risk factor for cardiovascular events (24, 25). Increasing evidence suggests that visceral fat poses a greater health risk than subcutaneous fat due to its stronger association with disease (26, 27). In support of this, a study by Kuk et al. (28) found that visceral fat, as measured by computed tomography, was a significant and independent predictor of all-cause mortality, in contrast to subcutaneous and liver fat. Despite this, a simple and effective proxy for visceral obesity is still needed. Emerging evidence indicates that the BRI, a newer anthropometric measure, more accurately reflects visceral fat compared to traditional metrics like BMI (5). Theoretically, BRI models the human body as an ellipse, with height as the long axis and waist circumference as the short axis, and is calculated as the eccentricity of this ellipse (5). Therefore, BRI may serve as a more effective anthropometric measure for assessing abdominal adiposity.

Although the idea that BRI can estimate the percentages of total and regional fat may be plausible and appealing, evidence on the association between BRI and disease is sparse, particularly in non-Western populations. In this study, utilizing data from CHARLS, we assessed the temporal trends of the BRI in the Chinese population from 2011 to 2015. The study found a generally stable trend in BRI over this period, with a noticeable increase between 2011 and 2013. This rise likely reflects broader trends in obesity and overweight observed globally, particularly in China, where rapid economic development and urbanization have led to significant changes in diet and lifestyle. Specifically, the increase from 2011 to 2013 may be attributed to factors such as the westernization of diets, increased consumption of high-calorie foods, and reduced physical activity levels. It is important to note that while BRI increased during this period, the rise was not as pronounced as the escalation seen in Body Mass Index (BMI) in other studies, possibly because BRI offers a more precise measure of body fat distribution, making it less sensitive to short-term fluctuations in weight. From 2013 to 2015, BRI tended to stabilize, which may be due to improvements in public health awareness and national-level initiatives aimed at combating obesity. These changes could have led to a slowing in the rate of weight gain, particularly as more people became aware of the long-term health risks associated with obesity. Additionally, the stabilization might reflect changes in China's socioeconomic structure and urbanization, which could have influenced diet and lifestyle factors, potentially reducing the rapid increase in BRI. The study also revealed interesting demographic differences in BRI trends. Women consistently had higher BRI values than men, likely due to differences in body fat distribution and hormonal influences on fat storage (29). Additionally, BRI increased with age, high BRI in individuals older than 60 years may be indicative of adipose tissue senescence and dysfunction (30, 31). This aligns with studies from other populations, such as those conducted in the U.S., where older adults and women have shown similar trends (14). In addition, the BRI is higher in urban China due to factors like high-calorie diets, sedentary lifestyles, and less physical activity (32, 33).

Our study also identifies risk factors associated with variations in BRI. The multivariable analysis revealed several key factors positively associated with higher BRI, including older age, alcohol consumption, elevated systolic and diastolic blood pressure, HbA1c, triglycerides, and the presence of chronic conditions such as diabetes and hypertension. On the other hand, male sex, rural residence, current smoking, and higher levels of fasting blood glucose and high-density lipoprotein cholesterol were negatively associated with BRI. These findings highlight the complexity of the factors influencing BRI and suggest that BRI is not only a measure of body fat distribution but is also closely linked to metabolic health. For example, the positive association between BRI and systolic and diastolic blood pressure aligns with the well-established link between abdominal obesity and hypertension (34, 35). Visceral fat, which BRI captures more effectively than BMI, is a known contributor to insulin resistance, which can lead to increased blood pressure and other metabolic disturbances (36, 37).

The relationship between BRI and CVD is increasingly recognized as an important area of study, however, there are still relatively few related studies. A study by Wu et al. (7) found a dose-dependent increase in cardiovascular events with higher BRI levels, especially in younger adults. Li et al. (38) found that BRI was superior to other indices for predicting CVD risk factors, among the Chinese. Our study also suggests a strong longitudinal association between baseline BRI and the onset of CVD, with higher BRI quintiles significantly correlating with increased risks for total CVD, stroke, and heart disease. Furthermore, sex and age appear to modify the relationship between BRI and CVD risk, with middle-aged participants and men showing a stronger association between high BRI and cardiovascular outcomes. In addition to the baseline BRI, our study also investigated the association of cumulative BRI with incident CVD, which was not examined previously. We also found an association between the cumulative BRI index and the risk of CVD. In addition, Zhang et al. (39) supported our conclusion by identifying three different BRI trajectories and finding a positive correlation between BRI trajectories and CVD incidence. However, Zhang et al.'s. study focuses on rural populations in Northeast China, our study utilizes data from the nationally representative CHARLS cohort, including both urban and rural populations. Additionally, our study incorporates mediation analyses to explore factors like hypertension and diabetes, providing deeper insight into the mechanisms underlying the relationship between BRI and CVD. One of the central reasons for BRI's strong predictive value in CVD is its ability to reflect visceral fat, which is metabolically active and contributes to endothelial dysfunction and atherosclerosis through the release of pro-inflammatory cytokines (40, 41). From clinical aspects, the accumulation of visceral fat was associated with more profound insulin resistance, then it is associated with cardiovascular risk even among participants with weight within the reference range (36, 37). This makes BRI superior to traditional measures like BMI, which only accounts for overall fat mass and does not differentiate where fat is distributed in the body. BRI's focus on fat distribution is particularly important because visceral fat is more closely linked to cardiovascular risk than subcutaneous fat.

Another important contribution of this study is its mediation analysis, which explored the mechanisms through which BRI influences CVD risk. The analysis revealed that hypertension was the most significant mediator, accounting for 20.69% of the BRI-associated increase in CVD risk. This finding underscores the central role of hypertension in the pathway linking visceral fat accumulation and cardiovascular disease. As visceral fat increases, so does the production of inflammatory markers and insulin resistance (40, 41), both of which contribute to the development of hypertension (42, 43). In turn, hypertension is a well-established risk factor for CVD (44), further amplifying the impact of high BRI on cardiovascular outcomes. Other significant mediators included dyslipidemia and HbA1c, both of which are closely tied to metabolic health. Dyslipidemia, characterized by abnormal levels of blood lipids such as triglycerides and cholesterol, is a common consequence of obesity and insulin resistance (45, 46). The mediation analysis showed that dyslipidemia mediated approximately 9.49% of the association between BRI and CVD, further highlighting the interconnectedness of these metabolic conditions. The significant role of HbA1c, a marker of long-term blood glucose levels, in mediating the relationship between BRI and CVD is also noteworthy. HbA1c reflects cumulative exposure to high blood glucose levels, which can lead to damage to blood vessels (47). This finding suggests that individuals with high BRI are not only at greater risk of developing metabolic conditions such as diabetes but are also more likely to experience the long-term consequences of poor glucose control, including CVD.

The findings of this study have several important implications for public health and clinical practice, particularly in China and other countries experiencing rapid increases in obesity and obesity-related diseases. First, BRI could serve as a more reliable measure for monitoring obesity-related health risks over time. Unlike BMI, which may fluctuate with changes in muscle mass or hydration status, BRI provides a more consistent assessment of visceral fat, which is closely linked to metabolic health and CVD risk. Second, the strong associations between BRI and CVD risk, particularly when mediated by hypertension, dyslipidemia, and HbA1c, suggest that targeting these metabolic conditions could help mitigate the cardiovascular risks associated with obesity. Public health interventions aimed at reducing hypertension, improving lipid profiles, and enhancing glucose control in individuals with high BRI could potentially reduce the burden of CVD in populations where obesity is on the rise. Third, the observed sex and age differences in BRI's association with CVD risk suggest that interventions should be tailored to specific demographic groups.

The strengths of this study included the large sample size, nationally representative cohort, and long-term follow-up. However, there are several limitations that should be acknowledged. First, the observational nature of the study precludes causal inferences between BRI and health outcomes. While the longitudinal design strengthens the evidence for a relationship between BRI and CVD, further research, particularly randomized controlled trials, is needed to confirm these findings and explore potential interventions. Second, the study was conducted in a Chinese population, which may limit the generalizability of the findings to other populations. Future research should explore BRI trends and associations in other regions, particularly in populations with different patterns of fat distribution. Three, consistent with other studies (48, 49), the diagnosis of CVD was self-reported due to logistical constraints. While medical records were not accessible in the CHARLS, other large-scale studies (50), such as the English Longitudinal Study of Aging, have demonstrated that self-reported incidents of coronary heart disease correlate well with medical records, exhibiting an accuracy of 77.5%. Finally, while the study identified key risk factors associated with BRI, it did not explore other potential factors that may influence body fat distribution, such as dietary habits, physical activity, and genetic predisposition. Future research should aim to identify additional determinants of BRI and explore how these factors interact to influence health outcomes.

## 5 Conclusions

This study provides strong evidence supporting the use of BRI as a predictor of obesity-related health risks, particularly cardiovascular disease, in a Chinese population. The slow upward trend in BRI observed across different demographic groups, coupled with the strong associations between higher BRI values and cardiovascular outcomes, highlights the importance of this metric in both clinical and public health settings. The findings also suggest that targeting hypertension and other metabolic conditions in individuals with high BRI could help reduce the burden of CVD. As obesity rates continue to rise globally, incorporating BRI into public health strategies could lead to more precise risk stratification and more effective prevention and treatment of obesity-related diseases.

## Data Availability

Publicly available datasets were analyzed in this study. This data can be found here: http://charls.pku.edu.cn/en.
