# Supplementary material for: Temporal trends, associated risk factors and longitudinal cardiovascular outcomes of body roundness among middle-aged and older Chinese adults: from the China Health and Retirement Longitudinal Study 2011–2018
Source: Front Nutr. 2025 Jan 24;12:1515067. doi: 10.3389/fnut.2025.1515067 (PMC11804525; doi:10.3389/fnut.2025.1515067)
Supplement: Supplementary file 1 [file Table_1.docx]

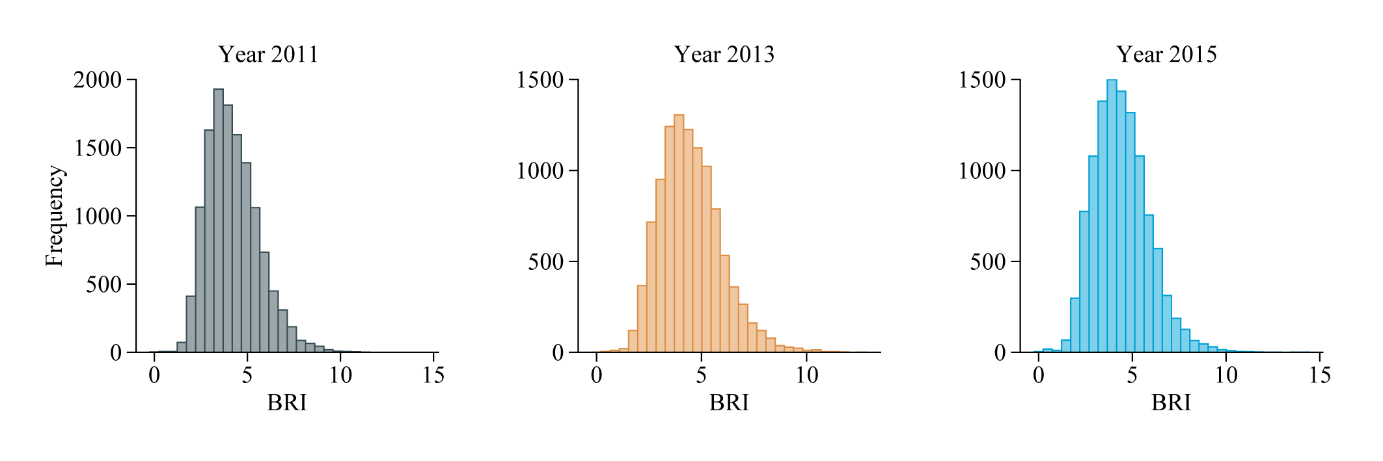


## Figure S1. Distribution of body roundness index at year 2011, 2013, and 2015

BRI, Body roundness index.

## Table S1. The missing number and rate of variables for risk factors analysis at year 2011

| Variables | Overall (n=12902) |
| --- | --- |
| Age (years) | 0 (0.0%) |
| Gender | 0 (0.0%) |
| Marital status | 0 (0.0%) |
| Residence | 0 (0.0%) |
| Education level | 0 (0.0%) |
| Smoking status | 0 (0.0%) |
| Drinking status | 71 (0.6%) |
| SBP (mmHg) | 18 (0.1%) |
| DBP (mmHg) | 130 (1.0%) |
| Diabetes | 131 (1.0%) |
| Hypertension | 29 (0.2%) |
| Dyslipidemia | 0 (0.0%) |
| Kidney disease | 69 (0.5%) |
| HbA1c (%) | 100 (0.8%) |
| FBG (mg/dl) | 3542 (27.5%) |
| TC (mg/dl) | 3619 (28.0%) |
| TG (mg/dl) | 3607 (28.0%) |
| HDL-C (mg/dl) | 3606 (27.9%) |
| LDL-C (mg/dl) | 3600 (27.9%) |
| hs-CRP (mg/l) | 3617 (28.0%) |
| eGFR (ml/min/1.73 m^2^) | 3599 (27.9%) |

DBP, Diastolic blood pressure; eGFR, Estimated glomerular filtration rate; FBG, Fasting plasma glucose; hs-CRP, High-density C-reactive protein; HbA1c, Glycated hemoglobin; HDL-C, High-density lipoprotein cholesterol; LDL-C, Low-density lipoprotein cholesterol; SBP, Systolic blood pressure; TC, Total cholesterol; TG, Triglyceride.

## Table S2. The missing number and rate of variables for longitudinal anlysis

at year 2011

| Variables | Overall (n=10525) |
| --- | --- |
| Age (years) | 0 (0.0%) |
| Gender | 0 (0.0%) |
| Marital status | 0 (0.0%) |
| Residence | 0 (0.0%) |
| Education level | 0 (0.0%) |
| Smoking status | 36 (0.3%) |
| Drinking status | 5 (0.0%) |
| SBP (mmHg) | 92 (0.9%) |
| DBP (mmHg) | 93 (0.9%) |
| Diabetes | 15 (0.1%) |
| Hypertension | 0 (0.0%) |
| Dyslipidemia | 41 (0.4%) |
| Kidney disease | 36 (0.3%) |
| HbA1c (%) | 2794 (26.5%) |
| FBG (mg/dl) | 2851 (27.1%) |
| TC (mg/dl) | 2845 (27.0%) |
| TG (mg/dl) | 2843 (27.0%) |
| HDL-C (mg/dl) | 2838 (27.0%) |
| LDL-C (mg/dl) | 2853 (27.1%) |
| hs-CRP (mg/l) | 2838 (27.0%) |
| eGFR (ml/min/1.73 m^2^) | 2854 (27.1%) |

DBP, Diastolic blood pressure; eGFR, Estimated glomerular filtration rate; FBG, Fasting plasma glucose; hs-CRP, High-density C-reactive protein; HbA1c, Glycated hemoglobin; HDL-C, High-density lipoprotein cholesterol; LDL-C, Low-density lipoprotein cholesterol; SBP, Systolic blood pressure; TC, Total cholesterol; TG, Triglyceride.

## Table S3. The missing number and rate of variables for cumulative anlysis at year 2011-2015

| Variables | Overall (n=7310) |
| --- | --- |
| Age (years) | 0 (0.0%) |
| Gender | 0 (0.0%) |
| Marital status | 0 (0.0%) |
| Residence | 0 (0.0%) |
| Education level | 0 (0.0%) |
| Smoking status | 0 (0.0%) |
| Drinking status | 24 (0.3%) |
| SBP (mmHg) | 5 (0.1%) |
| DBP (mmHg) | 64 (0.9%) |
| Diabetes | 65 (0.9%) |
| Hypertension | 8 (0.1%) |
| Dyslipidemia | 0 (0.0%) |
| Kidney disease | 21 (0.3%) |
| HbA1c (%) | 26 (0.4%) |
| FBG (mg/dl) | 1678 (23.0%) |
| TC (mg/dl) | 1705 (23.3%) |
| TG (mg/dl) | 1701 (23.3%) |
| HDL-C (mg/dl) | 1699 (23.2%) |
| LDL-C (mg/dl) | 1694 (23.2%) |
| hs-CRP (mg/l) | 1707 (23.4%) |
| eGFR (ml/min/1.73 m^2^) | 1695 (23.2%) |

DBP, Diastolic blood pressure; eGFR, Estimated glomerular filtration rate; FBG, Fasting plasma glucose; hs-CRP, High-density C-reactive protein; HbA1c, Glycated hemoglobin; HDL-C, High-density lipoprotein cholesterol; LDL-C, Low-density lipoprotein cholesterol; SBP, Systolic blood pressure; TC, Total cholesterol; TG, Triglyceride

## Table S4. Variance inflation factor of the variables

| Variables | Variance inflation factor | |
| --- | --- | --- |
|  | Before excluding the covariates with multicollinearity | After excluding the covariates with multicollinearity |
| Age (years) | 1.31 | 1.30 |
| Gender | 2.63 | 2.61 |
| Marital status | 1.06 | - |
| Residence | 1.09 | 1.09 |
| Education level | 1.41 | 1.39 |
| Smoking status | 1.99 | 1.99 |
| Drinking status | 1.49 | 1.49 |
| SBP (mmHg) | 3.16 | 3.14 |
| DBP (mmHg) | 2.39 | 2.38 |
| Diabetes | 1.71 | 1.71 |
| Hypertension | 1.99 | 1.99 |
| Dyslipidemia | 1.54 | 1.39 |
| Kidney disease | 1.02 | - |
| Stroke | 1.03 | 1.03 |
| Heart disease | 1.04 | 1.03 |
| HbA1c (%) | 1.75 | 1.73 |
| FBG (mg/dl) | 2.48 | 2.48 |
| TC (mg/dl) | 15.73 | - |
| TG (mg/dl) | 6.22 | 1.43 |
| HDL-C (mg/dl) | 3.55 | 1.35 |
| LDL-C (mg/dl) | 12.54 | - |
| hs-CRP (mg/l) | 1.02 | 1.01 |
| eGFR (ml/min/1.73 m^2^) | 1.28 | 1.26 |

DBP, Diastolic blood pressure; eGFR, Estimated glomerular filtration rate; FBG, Fasting plasma glucose; hs-CRP, High-density C-reactive protein; HbA1c, Glycated hemoglobin; HDL-C, High-density lipoprotein cholesterol; LDL-C, Low-density lipoprotein cholesterol; SBP, Systolic blood pressure; TC, Total cholesterol; TG, Triglyceride.

## Table S5. Baseline characteritics for risk factors analysis at year 2011 with complete data ^a^

| Characteritics | Overall (n=8999) |
| --- | --- |
| Age (years) | 59.23±9.31 |
| Age ≥60 years | 4016 (44.6%) |
| Men | 4197 (46.6%) |
| Married | 7512 (83.5%) |
| Rural residence | 5876 (65.3%) |
| Education level |  |
| No formal education | 2662 (29.6%) |
| Primary school | 3695 (41.1%) |
| Middle or high school | 2377 (26.4%) |
| College or above | 265 (2.9%) |
| Smoking status |  |
| Never | 5454 (60.6%) |
| Former | 799 (8.9%) |
| Current | 2746 (30.5%) |
| Drinking status |  |
| Never | 5307 (59.0%) |
| Former | 764 (8.5%) |
| Current | 2928 (32.5%) |
| SBP (mmHg) | 129.70±21.51 |
| DBP (mmHg) | 75.39±12.23 |
| Diabetes | 1487 (16.5%) |
| Hypertension | 3752 (41.7%) |
| Dyslipidemia | 4373 (48.6%) |
| Kidney disease | 538 (6.0%) |
| HbA1c (%) | 5.28±0.83 |
| FBG (mg/dl) | 110.36±37.23 |
| TC (mg/dl) | 193.69±38.28 |
| TG (mg/dl) | 105.32 (75.22, 153.99) |
| HDL-C (mg/dl) | 51.25±15.26 |
| LDL-C (mg/dl) | 116.73±35.04 |
| hs-CRP (mg/l) | 1.04 (0.55, 2.18) |
| eGFR (ml/min/1.73 m^2^) | 72.91 (53.79, 96.32) |

DBP, Diastolic blood pressure; eGFR, Estimated glomerular filtration rate; FBG, Fasting plasma glucose; hs-CRP, High-density C-reactive protein; HbA1c, Glycated hemoglobin; HDL-C, High-density lipoprotein cholesterol; LDL-C, Low-density lipoprotein cholesterol; SBP, Systolic blood pressure; TC, Total cholesterol; TG, Triglyceride.

^a^ Data were mean±SD, median (IQR) or n(%), unless otherwise specified.

## Table S6. Baseline characteritics for longitudinal anlysis at year 2011 with complete data ^a^

| Characteritics | Overall (n=7483) |
| --- | --- |
| Age (years) | 58.69±9.19 |
| Age ≥60 years | 3148 (42.1%) |
| Men | 3537 (47.3%) |
| Married | 6294 (84.1%) |
| Rural residence | 5002 (66.8%) |
| Education level |  |
| No formal education | 2220 (29.7%) |
| Primary school | 3051 (40.8%) |
| Middle or high school | 2006 (26.8%) |
| College or above | 206 (2.8%) |
| Smoking status |  |
| Never | 4541 (60.7%) |
| Former | 600 (8.0%) |
| Current | 2342 (31.3%) |
| Drinking status |  |
| Never | 4345 (58.1%) |
| Former | 597 (8.0%) |
| Current | 2541 (34.0%) |
| SBP (mmHg) | 128.92±21.10 |
| DBP (mmHg) | 75.11±12.11 |
| Diabetes | 1135 (15.2%) |
| Hypertension | 2872 (38.4%) |
| Dyslipidemia | 3470 (46.4%) |
| Kidney disease | 363 (4.9%) |
| HbA1c (%) | 5.26±0.81 |
| FBG (mg/dl) | 109.46±35.27 |
| TC (mg/dl) | 193.38±37.90 |
| TG (mg/dl) | 103.54 (74.34, 151.34) |
| HDL-C (mg/dl) | 51.65±15.25 |
| LDL-C (mg/dl) | 116.34±34.73 |
| hs-CRP (mg/l) | 0.99 (0.54, 2.09) |
| eGFR (ml/min/1.73 m^2^) | 72.87 (53.75, 95.95) |

DBP, Diastolic blood pressure; eGFR, Estimated glomerular filtration rate; FBG, Fasting plasma glucose; hs-CRP, High-density C-reactive protein; HbA1c, Glycated hemoglobin; HDL-C, High-density lipoprotein cholesterol; LDL-C, Low-density lipoprotein cholesterol; SBP, Systolic blood pressure; TC, Total cholesterol; TG, Triglyceride.

^a^ Data were mean±SD, median (IQR) or n(%), unless otherwise specified.

## Table S7. Baseline characteritics for cumulative anlysis at year 2011-2013 with complete data ^a^

| Characteritics | Overall (n=5473) |
| --- | --- |
| Age (years) | 58.83±9.11 |
| Age ≥60 years | 2352 (43.0%) |
| Men | 2577 (47.1%) |
| Married | 4644 (84.9%) |
| Rural residence | 3753 (68.6%) |
| Education level |  |
| No formal education | 1606 (29.3%) |
| Primary school | 2305 (42.1%) |
| Middle or high school | 1427 (26.1%) |
| College or above | 135 (2.5%) |
| Smoking status |  |
| Never | 3331 (60.9%) |
| Former | 440 (8.0%) |
| Current | 1702 (31.1%) |
| Drinking status |  |
| Never | 3192 (58.3%) |
| Former | 445 (8.1%) |
| Current | 1836 (33.5%) |
| SBP (mmHg) | 128.70±20.94 |
| DBP (mmHg) | 74.89±12.11 |
| Diabetes | 827 (15.1%) |
| Hypertension | 2074 (37.9%) |
| Dyslipidemia | 2493 (45.6%) |
| Kidney disease | 259 (4.7%) |
| HbA1c (%) | 5.25±0.79 |
| FBG (mg/dl) | 109.35±34.63 |
| TC (mg/dl) | 193.40±37.66 |
| TG (mg/dl) | 102.66 (73.46, 148.68) |
| HDL-C (mg/dl) | 52.01±15.37 |
| LDL-C (mg/dl) | 116.37±34.59 |
| hs-CRP (mg/l) | 0.96 (0.53, 1.99) |
| eGFR (ml/min/1.73 m^2^) | 72.58 (53.91, 95.91) |

DBP, Diastolic blood pressure; eGFR, Estimated glomerular filtration rate; FBG, Fasting plasma glucose; hs-CRP, High-density C-reactive protein; HbA1c, Glycated hemoglobin; HDL-C, High-density lipoprotein cholesterol; LDL-C, Low-density lipoprotein cholesterol; SBP, Systolic blood pressure; TC, Total cholesterol; TG, Triglyceride.

^a^ Data were mean±SD, median (IQR) or n(%), unless otherwise specified.

## Table S8. Baseline characteritics for longitudinal anlysis at year 2011 according to quintiles of body roundness index ^*^

| Characteritics | Quintile 1 [0.174, 3]  (n=2105) | Quintile 2 (3, 3.65]  (n=2105) | Quintile 3 (3.65, 4.36]  (n=2105) | Quintile 4 (4.36, 5.26]  (n=2106) | Quintile 5 (5.26, 14.3]  (n=2104) | P value |
| --- | --- | --- | --- | --- | --- | --- |
| Age (years) | 58.08±9.20 | 58.28±9.13 | 57.84±9.02 | 58.43±9.36 | 60.19±9.67 | <0.001 ^a^ |
| Age ≥60 years | 817 (38.8%) | 822 (39.0%) | 806 (38.3%) | 868 (41.2%) | 1022 (48.6%) | <0.001 ^b^ |
| Men | 1452 (69.0%) | 1265 (60.1%) | 1024 (48.6%) | 866 (41.1%) | 476 (22.6%) | <0.001 ^b^ |
| Married | 1758 (83.5%) | 1760 (83.6%) | 1748 (83.0%) | 1772 (84.1%) | 1713 (81.4%) | 0.163 ^b^ |
| Rural residence | 1573 (74.7%) | 1443 (68.6%) | 1362 (64.7%) | 1275 (60.5%) | 1236 (58.7%) | <0.001 ^b^ |
| Education level |  |  |  |  |  | <0.001 ^b^ |
| No formal education | 500 (23.8%) | 559 (26.6%) | 573 (27.2%) | 620 (29.4%) | 795 (37.8%) |  |
| Primary school | 951 (45.2%) | 845 (40.1%) | 836 (39.7%) | 816 (38.7%) | 797 (37.9%) |  |
| Middle or high school | 597 (28.4%) | 630 (29.9%) | 613 (29.1%) | 594 (28.2%) | 464 (22.1%) |  |
| College or above | 57 (2.7%) | 71 (3.4%) | 83 (3.9%) | 76 (3.6%) | 48 (2.3%) |  |
| Smoking status |  |  |  |  |  | <0.001 ^b^ |
| Never | 883 (41.9%) | 1087 (51.6%) | 1257 (59.7%) | 1421 (67.5%) | 1667 (79.2%) |  |
| Former | 160 (7.6%) | 171 (8.1%) | 184 (8.7%) | 184 (8.7%) | 129 (6.1%) |  |
| Current | 1062 (50.5%) | 847 (40.2%) | 664 (31.5%) | 501 (23.8%) | 308 (14.6%) |  |
| Drinking status |  |  |  |  |  | <0.001 ^b^ |
| Never | 1021 (48.5%) | 1093 (51.9%) | 1190 (56.5%) | 1300 (61.7%) | 1516 (72.1%) |  |
| Former | 167 (7.9%) | 155 (7.4%) | 186 (8.8%) | 166 (7.9%) | 141 (6.7%) |  |
| Current | 917 (43.6%) | 857 (40.7%) | 729 (34.6%) | 640 (30.4%) | 447 (21.2%) |  |
| SBP (mmHg) | 123.04±19.52 | 125.71±19.91 | 127.71±20.36 | 131.30±20.98 | 136.72±21.67 | <0.001 ^a^ |
| DBP (mmHg) | 72.28±11.78 | 73.58±11.94 | 74.76±11.86 | 76.70±11.95 | 78.61±11.73 | <0.001 ^a^ |
| Diabetes | 149 (7.1%) | 183 (8.7%) | 244 (11.6%) | 326 (15.5%) | 399 (19.0%) | <0.001 ^b^ |
| Hypertension | 497 (23.6%) | 623 (29.6%) | 734 (34.9%) | 947 (45.0%) | 1198 (56.9%) | <0.001 ^b^ |
| Dyslipidemia | 445 (21.1%) | 604 (28.7%) | 743 (35.3%) | 938 (44.5%) | 1081 (51.4%) | <0.001 ^b^ |
| Kidney disease | 107 (5.1%) | 103 (4.9%) | 107 (5.1%) | 93 (4.4%) | 87 (4.1%) | 0.492 ^b^ |
| HbA1c (%) | 5.12±0.60 | 5.14±0.65 | 5.20±0.79 | 5.28±0.76 | 5.38±0.89 | <0.001 ^a^ |
| FBG (mg/dl) | 103.35±27.15 | 104.04±28.01 | 107.50±33.62 | 111.20±37.48 | 112.91±37.00 | <0.001 ^a^ |
| TC (mg/dl) | 185.95±37.62 | 188.20±36.64 | 192.14±38.43 | 196.35±40.68 | 198.69±39.76 | <0.001 ^a^ |
| TG (mg/dl) | 82.31 (62.83, 119.47) | 91.15 (67.26, 130.10) | 100.00 (72.57, 139.83) | 110.62 (78.76, 161.07) | 121.25 (85.85, 181.43) | <0.001 ^c^ |
| HDL-C (mg/dl) | 57.48±15.82 | 55.00±15.28 | 53.53±15.75 | 50.24±15.16 | 48.46±14.47 | <0.001 ^a^ |
| LDL-C (mg/dl) | 109.95±32.86 | 112.76±33.11 | 115.05±34.12 | 117.81±36.01 | 119.70±36.91 | <0.001 ^a^ |
| hs-CRP (mg/l) | 0.80 (0.45, 1.71) | 0.83 (0.48, 1.78) | 0.93 (0.52, 1.97) | 1.06 (0.59, 2.14) | 1.33 (0.70, 2.59) | <0.001 ^c^ |
| eGFR (ml/min/1.73 m^2^) | 81.07 (60.18, 102.42) | 75.92 (57.21, 98.01) | 73.41 (53.54, 96.86) | 71.39 (53.13, 95.91) | 65.39 (46.83, 86.43) | <0.001 ^c^ |

DBP, Diastolic blood pressure; eGFR, Estimated glomerular filtration rate; FBG, Fasting plasma glucose; hs-CRP, High-density C-reactive protein; HbA1c, Glycated hemoglobin; HDL-C, High-density lipoprotein cholesterol; LDL-C, Low-density lipoprotein cholesterol; SBP, Systolic blood pressure; TC, Total cholesterol; TG, Triglyceride.

^*^ Data were mean±SD, median (IQR) or n(%), unless otherwise specified.

^a^ Calculated by one-way analysis of variance.

^b^ Calculated by Pearson's Chi-squared test.

^c^ Calculated by Kruskal-Wallis rank sum test.

## Table S9. Baseline characteritics according to quintiles of cumulative body roundness index ^*^

| Variable | Quintile 1 [2.29, 9.41]  (n=1462) | Quintile 3 (9.41, 11.4]  (n=1462) | Quintile 3 (11.4, 13.4]  (n=1462) | Quintile 4 (13.4, 16]  (n=1462) | Quintile 5 (16, 34.7]  (n=1462) | P value |
| --- | --- | --- | --- | --- | --- | --- |
| Age (years) | 58.79±8.93 | 58.29±8.88 | 58.77±9.56 | 58.30±8.86 | 59.75±9.41 | <0.001 ^a^ |
| Age ≥60 years | 626 (42.8%) | 582 (39.8%) | 625 (42.7%) | 611 (41.8%) | 685 (46.9%) | 0.003 ^b^ |
| Men | 1074 (73.5%) | 857 (58.6%) | 704 (48.2%) | 560 (38.3%) | 318 (21.8%) | <0.001 ^b^ |
| Married | 1220 (83.4%) | 1259 (86.1%) | 1204 (82.4%) | 1249 (85.4%) | 1215 (83.1%) | 0.024 ^b^ |
| Rural residence | 1129 (77.2%) | 1022 (69.9%) | 1004 (68.7%) | 911 (62.3%) | 878 (60.1%) | <0.001 ^b^ |
| Education level |  |  |  |  |  | <0.001 ^b^ |
| No formal education | 342 (23.4%) | 367 (25.1%) | 436 (29.8%) | 428 (29.3%) | 528 (36.1%) |  |
| Primary school | 691 (47.3%) | 635 (43.4%) | 580 (39.7%) | 599 (41.0%) | 571 (39.1%) |  |
| Middle or high school | 396 (27.1%) | 414 (28.3%) | 397 (27.2%) | 391 (26.7%) | 333 (22.8%) |  |
| College or above | 33 (2.3%) | 46 (3.1%) | 49 (3.4%) | 44 (3.0%) | 30 (2.1%) |  |
| Smoking status |  |  |  |  |  | <0.001 ^b^ |
| Never | 551 (37.7%) | 775 (53.0%) | 903 (61.8%) | 1006 (68.8%) | 1167 (79.8%) |  |
| Former | 127 (8.7%) | 121 (8.3%) | 126 (8.6%) | 120 (8.2%) | 83 (5.7%) |  |
| Current | 784 (53.6%) | 566 (38.7%) | 433 (29.6%) | 336 (23.0%) | 212 (14.5%) |  |
| Drinking status |  |  |  |  |  | <0.001 ^b^ |
| Never | 662 (45.3%) | 759 (51.9%) | 839 (57.4%) | 938 (64.2%) | 1076 (73.6%) |  |
| Former | 135 (9.2%) | 98 (6.7%) | 135 (9.2%) | 112 (7.7%) | 96 (6.6%) |  |
| Current | 665 (45.5%) | 605 (41.4%) | 488 (33.4%) | 412 (28.2%) | 290 (19.8%) |  |
| SBP (mmHg) | 122.61±18.89 | 125.47±20.29 | 128.08±20.11 | 130.47±20.38 | 136.29±21.49 | <0.001 ^a^ |
| DBP (mmHg) | 71.35±11.28 | 73.53±12.08 | 74.43±11.61 | 76.23±11.75 | 78.85±11.89 | <0.001 ^a^ |
| Diabetes | 116 (7.9%) | 136 (9.3%) | 194 (13.3%) | 209 (14.3%) | 279 (19.1%) | <0.001 ^b^ |
| Hypertension | 319 (21.8%) | 426 (29.1%) | 527 (36.0%) | 628 (43.0%) | 828 (56.6%) | <0.001 ^b^ |
| Dyslipidemia | 311 (21.3%) | 426 (29.1%) | 533 (36.5%) | 662 (45.3%) | 766 (52.4%) | <0.001 ^b^ |
| Kidney disease | 81 (5.5%) | 75 (5.1%) | 63 (4.3%) | 62 (4.2%) | 66 (4.5%) | 0.388 ^b^ |
| HbA1c (%) | 5.13±0.65 | 5.15±0.62 | 5.19±0.72 | 5.28±0.78 | 5.39±0.89 | <0.001 ^a^ |
| FBG (mg/dl) | 103.87±28.13 | 104.57±28.17 | 107.92±31.95 | 110.65±38.41 | 113.09±36.47 | <0.001 ^a^ |
| TC (mg/dl) | 185.83±36.90 | 188.89±36.08 | 190.91±38.44 | 196.80±40.62 | 199.55±40.22 | <0.001 ^a^ |
| TG (mg/dl) | 80.54 (61.95, 110.62) | 90.27 (66.38, 129.21) | 101.78 (71.68, 141.60) | 108.86 (77.88, 162.62) | 122.13 (87.61, 182.31) | <0.001 ^c^ |
| HDL-C (mg/dl) | 58.08±15.91 | 55.31±15.21 | 52.45±15.47 | 50.65±15.42 | 48.32±14.16 | <0.001 ^a^ |
| LDL-C (mg/dl) | 109.74±32.01 | 113.52±32.93 | 114.38±34.32 | 118.14±36.35 | 120.33±36.61 | <0.001 ^a^ |
| hs-CRP (mg/l) | 0.81 (0.44, 1.80) | 0.78 (0.47, 1.64) | 0.91 (0.52, 1.86) | 1.05 (0.58, 1.99) | 1.33 (0.69, 2.62) | <0.001 ^c^ |
| eGFR (ml/min/1.73 m^2^) | 81.90 (60.97, 103.37) | 74.44 (56.01, 97.27) | 71.29 (53.68, 94.93) | 71.10 (52.72, 94.96) | 65.25 (47.90, 86.74) | <0.001 ^c^ |

DBP, Diastolic blood pressure; eGFR, Estimated glomerular filtration rate; FBG, Fasting plasma glucose; hs-CRP, High-density C-reactive protein; HbA1c, Glycated hemoglobin; HDL-C, High-density lipoprotein cholesterol; LDL-C, Low-density lipoprotein cholesterol; SBP, Systolic blood pressure; TC, Total cholesterol; TG, Triglyceride.

^*^ Data were mean±SD, median (IQR) or n(%), unless otherwise specified.

^a^ Calculated by one-way analysis of variance.

^b^ Calculated by Pearson's Chi-squared test.

^c^ Calculated by Kruskal-Wallis rank sum test.

## Table S10. Multivariable analysis for factors associated with body roundness index in 2011 in subpopulations of 8 999 participants with complete data

| Factors | β (95% CI) | P value |
| --- | --- | --- |
| Age (years) |  |  |
| <60 | 0 (Reference) |  |
| ≥60 | 0.18 (0.13 to 0.24) | <0.001 |
| Gender |  |  |
| Women | 0 (Reference) |  |
| Men | -0.79 (-0.87 to -0.71) | <0.001 |
| Residence |  |  |
| Urban | 0 (Reference) |  |
| Rural | -0.18 (-0.23 to -0.13) | <0.001 |
| Education level |  |  |
| No formal education | 0 (Reference) |  |
| Primary school | -0.03 (-0.10 to 0.03) | 0.292 |
| Middle or high school | -0.01 (-0.09 to 0.06) | 0.742 |
| College or above | -0.15 (-0.31 to 0.00) | 0.057 |
| Smoking status |  |  |
| Never | 0 (Reference) |  |
| Former | 0.02 (-0.08 to 0.12) | 0.741 |
| Current | -0.27 (-0.35 to -0.20) | <0.001 |
| Drinking status |  |  |
| Never | 0 (Reference) |  |
| Former | 0.08 (-0.02 to 0.17) | 0.111 |
| Current | 0.10 (0.04 to 0.16) | 0.002 |
| SBP (mmHg), per 1-SD increment | 0.06 (0.02 to 0.10) | 0.005 |
| DBP (mmHg) , per 1-SD increment | 0.10 (0.07 to 0.14) | <0.001 |
| Diabetes |  |  |
| No | 0 (Reference) |  |
| Yes | 0.22 (0.13 to 0.31) | <0.001 |
| Hypertension |  |  |
| No | 0 (Reference) |  |
| Yes | 0.34 (0.27 to 0.40) | <0.001 |
| Dyslipidemia |  |  |
| No | 0 (Reference) |  |
| Yes | 0.26 (0.20 to 0.32) | <0.001 |
| Stroke |  |  |
| No | 0 (Reference) |  |
| Yes | 0.13 (0.04 to 0.21) | 0.004 |
| Heart disease |  |  |
| No | 0 (Reference) |  |
| Yes | 0.13 (0.07 to 0.19) | <0.001 |
| HbA1c (%), per 1-SD increment | 0.15 (0.12 to 0.19) | <0.001 |
| FBG (mg/dl), per 1-SD increment | -0.09 (-0.13 to -0.05) | <0.001 |
| TG (mg/dl), per 1-SD increment | 0.06 (0.03 to 0.09) | <0.001 |
| HDL-C (mg/dl), per 1-SD incremen | -0.24 (-0.27 to -0.21) | <0.001 |
| hs-CRP (mg/l), per 1-SD increment | 0.02 (-0.00 to 0.05) | 0.057 |
| eGFR (ml/min/1.73 m^2^), per 1-SD increment | 0.00 (-0.02 to 0.03) | 0.780 |

Abbreviations: CI, Confidence interval; DBP, Diastolic blood pressure; eGFR, Estimated glomerular filtration rate; FBG, Fasting plasma glucose; hs-CRP, High-density C-reactive protein; HbA1c, Glycated hemoglobin; HDL-C, High-density lipoprotein cholesterol; SBP, Systolic blood pressure; TG, Triglyceride.

## Table S11. Longitudinal associations between baseline BRI and CVD in subpopulations of 7 483 participants with complete data

| Outcomes | No. of event / total | Model 1 ^a^ | |  | Model 2 ^b^ | |  | Model 3 ^c^ | |
| --- | --- | --- | --- | --- | --- | --- | --- | --- | --- |
|  |  | HR (95% CI) | P value |  | HR (95% CI) | P value |  | HR (95% CI) | P value |
| CVD |  |  |  |  |  |  |  |  |  |
| BRI, quintiles |  |  |  |  |  |  |  |  |  |
| Quintile 1 | 192 / 1447 | 1 (Reference) |  |  | 1 (Reference) |  |  | 1 (Reference) |  |
| Quintile 2 | 267 / 1493 | 1.38 (1.15–1.66) | 0.001 |  | 1.38 (1.14–1.66) | 0.001 |  | 1.32 (1.09–1.59) | 0.004 |
| Quintile 3 | 269 / 1482 | 1.38 (1.14–1.66) | 0.001 |  | 1.35 (1.12–1.63) | 0.002 |  | 1.24 (1.03–1.50) | 0.025 |
| Quintile 4 | 312 / 1516 | 1.52 (1.27–1.83) | <0.001 |  | 1.49 (1.24–1.79) | <0.001 |  | 1.27 (1.05–1.54) | 0.013 |
| Quintile 5 | 429 / 1545 | 2.01 (1.68–2.41) | <0.001 |  | 1.99 (1.66–2.38) | <0.001 |  | 1.57 (1.29–1.90) | <0.001 |
| BRI, per 1-SD increment | 1469 / 7483 | 1.23 (1.17–1.29) | <0.001 |  | 1.22 (1.16–1.29) | <0.001 |  | 1.13 (1.07–1.19) | <0.001 |
| Stroke |  |  |  |  |  |  |  |  |  |
| BRI, quintiles |  |  |  |  |  |  |  |  |  |
| Quintile 1 | 59 / 1447 | 1 (Reference) |  |  | 1 (Reference) |  |  | 1 (Reference) |  |
| Quintile 2 | 66 / 1493 | 1.13 (0.80–1.61) | 0.486 |  | 1.14 (0.80–1.62) | 0.461 |  | 1.05 (0.74–1.50) | 0.781 |
| Quintile 3 | 99 / 1482 | 1.80 (1.30–2.49) | <0.001 |  | 1.80 (1.30–2.49) | <0.001 |  | 1.49 (1.07–2.08) | 0.017 |
| Quintile 4 | 112 / 1516 | 1.99 (1.45–2.74) | <0.001 |  | 2.01 (1.46–2.78) | <0.001 |  | 1.44 (1.03–2.02) | 0.033 |
| Quintile 5 | 143 / 1545 | 2.59 (1.88–3.56) | <0.001 |  | 2.60 (1.88–3.58) | <0.001 |  | 1.61 (1.14–2.28) | 0.006 |
| BRI, per 1-SD increment | 479 / 7483 | 1.36 (1.25–1.48) | <0.001 |  | 1.36 (1.25–1.48) | <0.001 |  | 1.16 (1.05–1.27) | 0.003 |
| Heart disease |  |  |  |  |  |  |  |  |  |
| BRI, quintiles |  |  |  |  |  |  |  |  |  |
| Quintile 1 | 144 / 1447 | 1 (Reference) |  |  | 1 (Reference) |  |  | 1 (Reference) |  |
| Quintile 2 | 217 / 1493 | 1.46 (1.18–1.80) | <0.001 |  | 1.45 (1.17–1.79) | 0.001 |  | 1.41 (1.14–1.74) | 0.002 |
| Quintile 3 | 201 / 1482 | 1.31 (1.05–1.62) | 0.015 |  | 1.27 (1.03–1.58) | 0.029 |  | 1.21 (0.97–1.50) | 0.093 |
| Quintile 4 | 224 / 1516 | 1.36 (1.10–1.69) | 0.004 |  | 1.32 (1.06–1.63) | 0.012 |  | 1.19 (0.96–1.49) | 0.118 |
| Quintile 5 | 336 / 1545 | 1.90 (1.55–2.33) | <0.001 |  | 1.86 (1.51–2.28) | <0.001 |  | 1.59 (1.28–1.99) | <0.001 |
| BRI, per 1-SD increment | 1122 / 7483 | 1.19 (1.12–1.26) | <0.001 |  | 1.18 (1.11–1.25) | <0.001 |  | 1.12 (1.05–1.19) | 0.001 |

Abbreviations: BRI, Body roundness index; CVD, Cardiovascular disease; HR, hazard ratio.

^a^ Adjusted for age and gender.

^b^ Adjusted for age, gender, marital status, residence, education level, smoking status, and drinking status.

^c^ Adjusted for age, gender, marital status, residence, education level, smoking status, drinking status, systole blood pressure, diastolic blood pressure, diabetes, hypertension, dyslipidemia, kidney disease, glycated hemoglobin, fasting plasma glucose, total cholesterol, triglycerides, high-density lipoprotein cholesterol, low-density lipoprotein cholesterol, high-sensitivity c-reactive protein, and estimated glomerular filtration rate.

## Table S12. Associations between cumulative BRI and CVD in subpopulations of 5 473 participants with complete data

| Outcomes | No. of event / total | Model 1 ^a^ | |  | Model 2 ^b^ | |  | Model 3 ^c^ | |
| --- | --- | --- | --- | --- | --- | --- | --- | --- | --- |
|  |  | HR (95% CI) | P value |  | HR (95% CI) | P value |  | HR (95% CI) | P value |
| CVD |  |  |  |  |  |  |  |  |  |
| Cumulative, quintiles |  |  |  |  |  |  |  |  |  |
| Quintile 1 | 126 / 1065 | 1 (Reference) |  |  | 1 (Reference) |  |  | 1 (Reference) |  |
| Quintile 2 | 171 / 1111 | 1.33 (1.06–1.68) | 0.015 |  | 1.34 (1.06–1.69) | 0.014 |  | 1.28 (1.01–1.61) | 0.040 |
| Quintile 3 | 183 / 1087 | 1.42 (1.13–1.78) | 0.003 |  | 1.40 (1.11–1.77) | 0.004 |  | 1.28 (1.01–1.62) | 0.039 |
| Quintile 4 | 215 / 1096 | 1.69 (1.35–2.12) | <0.001 |  | 1.68 (1.34–2.11) | <0.001 |  | 1.47 (1.16–1.86) | 0.001 |
| Quintile 5 | 270 / 1114 | 1.97 (1.58–2.47) | <0.001 |  | 1.96 (1.56–2.46) | <0.001 |  | 1.57 (1.23–2.00) | <0.001 |
| Cumulative BRI, per 1-SD increment | 965 / 5473 | 1.24 (1.17–1.32) | <0.001 |  | 1.24 (1.16–1.32) | <0.001 |  | 1.15 (1.07–1.23) | <0.001 |
| Stroke |  |  |  |  |  |  |  |  |  |
| Cumulative BRI, quintiles |  |  |  |  |  |  |  |  |  |
| Quintile 1 | 42 / 1065 | 1 (Reference) |  |  | 1 (Reference) |  |  | 1 (Reference) |  |
| Quintile 2 | 47 / 1111 | 1.15 (0.75–1.74) | 0.524 |  | 1.17 (0.77–1.77) | 0.473 |  | 1.05 (0.69–1.60) | 0.813 |
| Quintile 3 | 60 / 1087 | 1.50 (1.01–2.23) | 0.045 |  | 1.53 (1.02–2.28) | 0.039 |  | 1.22 (0.81–1.84) | 0.331 |
| Quintile 4 | 77 / 1096 | 2.06 (1.40–3.02) | <0.001 |  | 2.13 (1.44–3.15) | <0.001 |  | 1.54 (1.03–2.31) | 0.037 |
| Quintile 5 | 90 / 1114 | 2.37 (1.61–3.48) | <0.001 |  | 2.44 (1.65–3.61) | <0.001 |  | 1.50 (0.99–2.30) | 0.058 |
| Cumulative BRI, per 1-SD increment | 316 / 5473 | 1.36 (1.22–1.51) | <0.001 |  | 1.36 (1.22–1.51) | <0.001 |  | 1.17 (1.04–1.32) | 0.009 |
| Heart disease |  |  |  |  |  |  |  |  |  |
| Cumulative BRI, quintiles |  |  |  |  |  |  |  |  |  |
| Quintile 1 | 91 / 1065 | 1 (Reference) |  |  | 1 (Reference) |  |  | 1 (Reference) |  |
| Quintile 2 | 137 / 1111 | 1.43 (1.10–1.87) | 0.008 |  | 1.42 (1.09–1.86) | 0.010 |  | 1.39 (1.06–1.81) | 0.017 |
| Quintile 3 | 140 / 1087 | 1.42 (1.09–1.86) | 0.009 |  | 1.40 (1.07–1.83) | 0.014 |  | 1.33 (1.01–1.75) | 0.040 |
| Quintile 4 | 154 / 1096 | 1.55 (1.19–2.02) | 0.001 |  | 1.51 (1.16–1.98) | 0.002 |  | 1.39 (1.06–1.84) | 0.019 |
| Quintile 5 | 202 / 1114 | 1.82 (1.40–2.36) | <0.001 |  | 1.79 (1.37–2.32) | <0.001 |  | 1.55 (1.17–2.06) | 0.003 |
| Cumulative BRI, per 1-SD increment | 724 / 5473 | 1.18 (1.10–1.27) | <0.001 |  | 1.17 (1.09–1.26) | <0.001 |  | 1.11 (1.03–1.21) | 0.009 |

Abbreviations: BRI, Body roundness index; CVD, Cardiovascular disease; HR, hazard ratio.

^a^ Adjusted for age and gender.

^b^ Adjusted for age, gender, marital status, residence, education level, smoking status, and drinking status.

^c^ Adjusted for age, gender, marital status, residence, education level, smoking status, drinking status, systole blood pressure, diastolic blood pressure, diabetes, hypertension, dyslipidemia, kidney disease, glycated hemoglobin, fasting plasma glucose, total cholesterol, triglycerides, high-density lipoprotein cholesterol, low-density lipoprotein cholesterol, high-sensitivity c-reactive protein, and estimated glomerular filtration rate.

## Table S13. Mediated proportion on the associations between body roundness index and CVD by different factors in subpopulations of 7483 participants with complete data

| Meditors | Associations, HR (95% CI) ^a^ | | | Proportion mediated,  % (95% CI) |
| --- | --- | --- | --- | --- |
|  | Total association | Direct association | Indirect association |  |
| CVD |  |  |  |  |
| Diabetes | 1.16 (1.12 to 1.20) | 1.16 (1.11 to 1.20) | 1.00 (1.00 to 1.00) | 0.93 (-0.70 to 2.56) |
| Hypertension | 1.14 (1.10 to 1.18) | 1.11 (1.07 to 1.16) | 1.02 (1.02 to 1.03) | 17.44 (10.17 to 24.71) |
| Dyslipidemia | 1.15 (1.11 to 1.20) | 1.14 (1.09 to 1.18) | 1.01 (1.01 to 1.02) | 10.79 (4.87 to 16.70) |
| Kidney disease | 1.16 (1.12 to 1.20) | 1.16 (1.12 to 1.20) | 1.00 (1.00 to 1.00) | -0.48 (-2.27 to 1.32) |
| HbA1c (%) | 1.16 (1.12 to 1.20) | 1.15 (1.11 to 1.20) | 1.00 (1.00 to 1.01) | 3.49 (-0.29 to 7.27) |
| FBG (mg/dl) | 1.16 (1.12 to 1.20) | 1.16 (1.11 to 1.20) | 1.00 (1.00 to 1.01) | 1.99 (-1.37 to 5.36) |
| hs-CRP (mg/l) | 1.16 (1.12 to 1.20) | 1.16 (1.12 to 1.20) | 1.00 (1.00 to 1.00) | 0.25 (-0.77 to 1.28) |
| eGFR (ml/min/1.73 m^2^) | 1.16 (1.12 to 1.20) | 1.16 (1.12 to 1.20) | 1.00 (1.00 to 1.00) | -0.20 (-1.06 to 0.67) |
| Stroke |  |  |  |  |
| Diabetes | 1.25 (1.17 to 1.33) | 1.24 (1.17 to 1.33) | 1.00 (1.00 to 1.01) | 1.33 (-0.70 to 3.36) |
| Hypertension | 1.22 (1.15 to 1.30) | 1.17 (1.10 to 1.25) | 1.04 (1.03 to 1.05) | 21.54 (11.88 to 31.21) |
| Dyslipidemia | 1.24 (1.16 to 1.32) | 1.21 (1.13 to 1.29) | 1.03 (1.01 to 1.04) | 13.17 (5.46 to 20.89) |
| Kidney disease | 1.25 (1.18 to 1.33) | 1.25 (1.18 to 1.33) | 1.00 (1.00 to 1.00) | -0.01 (-0.27 to 0.25) |
| HbA1c (%) | 1.25 (1.18 to 1.33) | 1.24 (1.17 to 1.32) | 1.01 (1.00 to 1.02) | 4.41 (0.25 to 8.57) |
| FBG (mg/dl) | 1.25 (1.18 to 1.33) | 1.24 (1.17 to 1.32) | 1.01 (1.00 to 1.02) | 4.57 (1.09 to 8.06) |
| hs-CRP (mg/l) | 1.25 (1.18 to 1.33) | 1.25 (1.17 to 1.33) | 1.00 (1.00 to 1.00) | 0.68 (-0.38 to 1.74) |
| eGFR (ml/min/1.73 m^2^) | 1.25 (1.18 to 1.33) | 1.25 (1.17 to 1.33) | 1.00 (1.00 to 1.00) | 0.05 (-0.66 to 0.76) |
| Heart disease |  |  |  |  |
| Diabetes | 1.13 (1.08 to 1.18) | 1.13 (1.08 to 1.18) | 1.00 (1.00 to 1.00) | 1.02 (-1.24 to 3.28) |
| Hypertension | 1.11 (1.06 to 1.16) | 1.09 (1.04 to 1.14) | 1.02 (1.01 to 1.02) | 17.67 (7.48 to 27.86) |
| Dyslipidemia | 1.12 (1.08 to 1.17) | 1.11 (1.06 to 1.16) | 1.01 (1.00 to 1.02) | 9.30 (1.59 to 17.02) |
| Kidney disease | 1.13 (1.08 to 1.18) | 1.13 (1.08 to 1.18) | 1.00 (1.00 to 1.00) | -0.77 (-3.67 to 2.13) |
| HbA1c (%) | 1.13 (1.08 to 1.18) | 1.13 (1.08 to 1.17) | 1.00 (1.00 to 1.01) | 2.92 (-2.34 to 8.18) |
| FBG (mg/dl) | 1.13 (1.08 to 1.18) | 1.13 (1.08 to 1.18) | 1.00 (0.99 to 1.01) | 0.45 (-4.42 to 5.33) |
| hs-CRP (mg/l) | 1.13 (1.08 to 1.18) | 1.13 (1.08 to 1.18) | 1.00 (1.00 to 1.00) | 0.02 (-1.48 to 1.52) |
| eGFR (ml/min/1.73 m^2^) | 1.13 (1.08 to 1.18) | 1.13 (1.08 to 1.18) | 1.00 (1.00 to 1.00) | -0.35 (-1.67 to 0.98) |

Abbreviations: CVD, Cardiovascular disease; eGFR, Estimated glomerular filtration rate; FBG, Fasting plasma glucose; hs-CRP, High-density C-reactive protein; HbA1c, Glycated hemoglobin; HR, hazard ratio.

^a^ Adjusted for age, gender, marital status, residence, education level, smoking status, and drinking status.
